# Genetics of Venous Thrombosis: Insights from a New Genome Wide Association Study

**DOI:** 10.1371/journal.pone.0025581

**Published:** 2011-09-27

**Authors:** Marine Germain, Noémie Saut, Nicolas Greliche, Christian Dina, Jean-Charles Lambert, Claire Perret, William Cohen, Tiphaine Oudot-Mellakh, Guillemette Antoni, Marie-Christine Alessi, Diana Zelenika, François Cambien, Laurence Tiret, Marion Bertrand, Anne-Marie Dupuy, Luc Letenneur, Mark Lathrop, Joseph Emmerich, Philippe Amouyel, David-Alexandre Trégouët, Pierre-Emmanuel Morange

**Affiliations:** 1 INSERM UMR_S 937; ICAN Institute, Université Pierre et Marie Curie, Paris 6; Paris, France; 2 INSERM, UMR_S 626, Marseille, France; Université de la Méditerranée, Marseille, France; 3 INSERM UMR_S 915; CNRS ERL3147; Institut du Thorax; Nantes, France; 4 INSERM U744, Lille, France; Institut Pasteur de Lille, Lille, France Université de Lille Nord de France, Lille, France; 5 Commissariat à l'Energie Atomique, Institut de Génomique, Centre National de Génotypage, Evry, France; 6 INSERM UMR_S 708, Université Pierre et Marie Curie (UPMC, Paris 6), Paris, France; 7 INSERM U888, Hôpital La Colombière, Montpellier, France; 8 INSERM, U897, Bordeaux, France; Université Victor Segalen, Bordeaux, France; 9 INSERM U765, médecine vasculaire - HTA, hôpital européen Georges-Pompidou, Université Paris-Descartes, Paris, France; 10 CHRU de Lille, Lille, France; Universitätsklinikum Schleswig-Holstein - Campus Luebeck, Germany

## Abstract

**Background:**

Venous Thrombosis (VT) is a common multifactorial disease associated with a major public health burden. Genetics factors are known to contribute to the susceptibility of the disease but how many genes are involved and their contribution to VT risk still remain obscure. We aimed to identify genetic variants associated with VT risk.

**Methodology/Principal Findings:**

We conducted a genome-wide association study (GWAS) based on 551,141 SNPs genotyped in 1,542 cases and 1,110 controls. Twelve SNPs reached the genome-wide significance level of 2.0×10^−8^ and encompassed four known VT-associated loci, *ABO*, *F5*, *F11* and *FGG*. By means of haplotype analyses, we also provided novel arguments in favor of a role of *HIVEP1*, *PROCR* and *STAB2*, three loci recently hypothesized to participate in the susceptibility to VT. However, no novel VT-associated loci came out of our GWAS. Using a recently proposed statistical methodology, we also showed that common variants could explain about 35% of the genetic variance underlying VT susceptibility among which 3% could be attributable to the main identified VT loci. This analysis additionally suggested that the common variants left to be identified are not uniformly distributed across the genome and that chromosome 20, itself, could contribute to ∼7% of the total genetic variance.

**Conclusions/Significance:**

This study might also provide a valuable source of information to expand our understanding of biological mechanisms regulating quantitative biomarkers for VT.

## Introduction

Venous thrombosis (VT) is a common multifactorial disease affecting two individuals out of one thousand a year and associated with a mortality rate of 10% [1]. Recurrence risk of VT is about 6% a year, and post-thrombotic disease occurs within the next 5 years following a VT event in about 25% of patients [2]. It has been reported that 25,000 individuals die from the consequences of VT each year in England [3] and that the disease has a substantial economic costs [4], [5]. Despite these striking elements, venous thrombosis can be considered as the Cinderella of genetic research on thrombotic disorders compared to arterial and cerebral thrombosis. Even though genetic factors are estimated to explain up to 60% of the VT heritability [6], [7], VT genetics has not benefit a lot from the genome wide association study (GWAS) revolution. While several GWAS and meta-analysis of GWAS have been conducted for arterial and cerebral thrombosis on thousands of individuals [8]–[14], only one GWAS on VT has been reported so far [15], and on a rather small sample of 419 cases and 1,228 controls. Before this GWAS was carried out, well-established susceptibility genes for VT were *SERPINC1*, *PROC*, *PROS1*, *FII*, *FGG*, *FV* and *ABO* [16]. The latter two loci were the only genomic regions that reached genome-wide statistical significance in the VT GWAS. Nevertheless, using additional strategies to assess the most promising associations generated by this GWAS, other VT-associated loci were robustly identified, *HIVEP1* [17], *C4BPA* [18], and *TC2N* [19]. Two additional VT-associated loci, *GP6* and *F11* [20], were also robustly identified through another large-scale association study, focusing mainly on non-synonymous polymorphisms.

**Figure 1:**
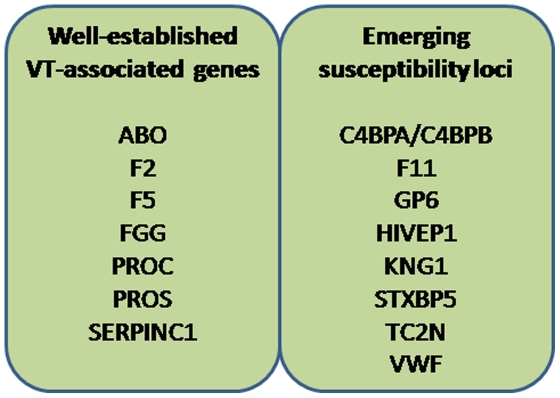
Known candidate loci for VT.

**Figure 2:**
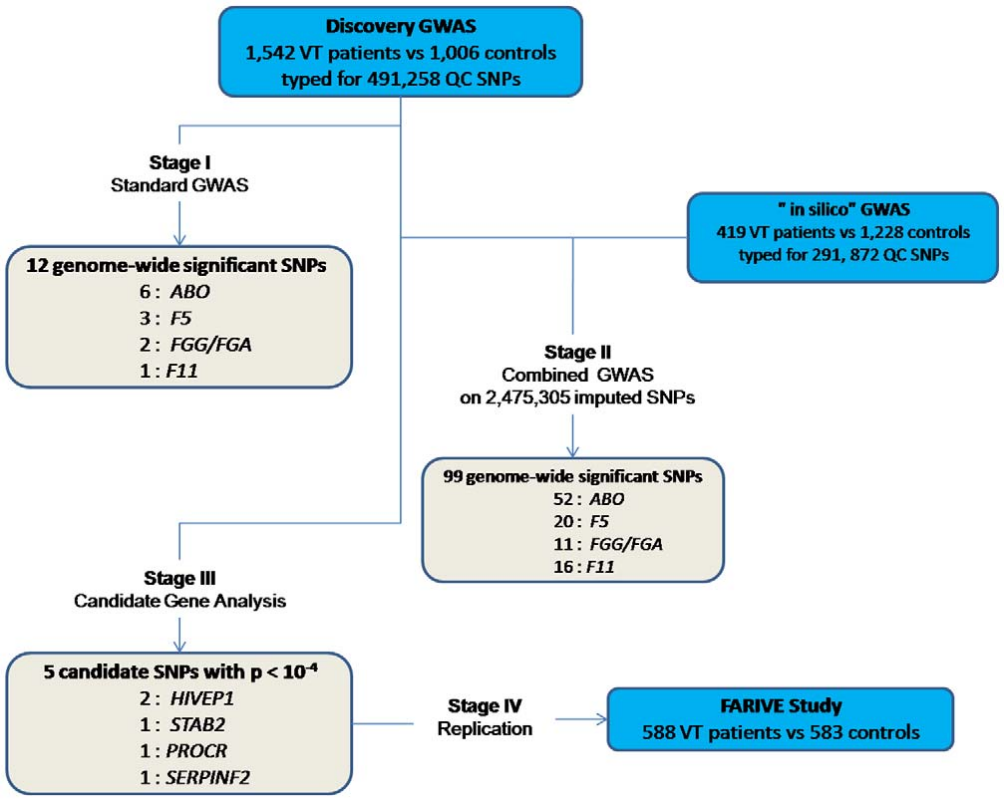
Main outlines of the adopted sequential GWAS strategy.

In our quest to identify novel susceptibility genes for VT beyond those already known (Figure 1), we report the results of a second GWAS based on a larger sample size (1,542 cases and 1,110 controls) and exploring a larger number of single nucleotide polymorphisms (SNPs) (551,141 vs 317,139 in the previous GWAS [15]). The overall sequential procedure of this work was summarized in Figure 2. A standard GWAS comparing VT patients participating in the MARTHA project [21] to healthy individuals from the Three-City Study (3C) [22] was first performed to identify genome-wide significant associations of SNPs with VT risk (stage I). Second, results from this GWAS were combined to those of our previously published GWAS on VT (referred to as “in silico GWAS” in the rest of the document) [15] to detect novel associations that would not have been declared significant at stage I. At this stage (stage II), both raw and imputed genotyped data analyses were carried out. In addition to this standard GWAS, we performed a candidate gene association analysis using less stringent statistical thresholds. SNPs demonstrating suggestive evidence of association with the disease were then planned to be further tested for replication in independent case-controls studies. A new estimate of the genetic variance associated with VT susceptibility was also derived using a novel methodology for GWAS data.

## Results

### Genome Wide Association analysis

#### Stage I

**Figure 3:**
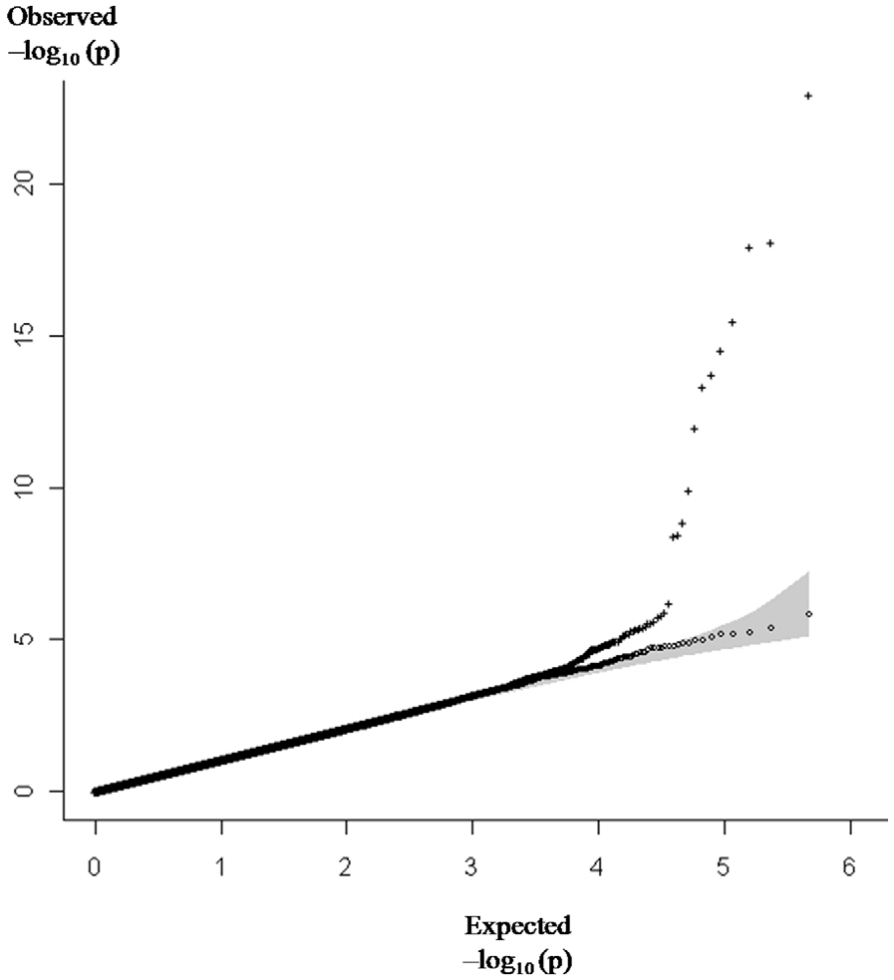
Quantile-Quantile plot representation of the GWAS results obtained from 491,258 studied SNPs. Q-Q plot derived from all SNP p-values is illustrated by + . The exclusion of 878 SNPs located within ±500 kb of the *ABO*, *F5*, *FGG* and *FXI* loci, the four main well-established VT-associated loci, lead to the Q-Q plot symbolized by ○ with its 95% confidence interval in shaded area.

A quantile-quantile (Q-Q) plot representation of the whole set of association results was compatible with what was expected under the assumption of no genetic association (Figure 3) and the corresponding genomic control (GC) value was 1.04. Among the 491,258 tested SNPs at this stage, twelve were significant at the fixed genome-wide threshold of p<2.0×10^−8^ (Table 1). These SNPs were located within *ABO*, *FGG*, *F5*, and *F11* loci, four well-established VT-associated genes. The *F5* and *ABO* hit SNPs included those already identified in our previous GWAS [15] while the *FGG* VT-associated SNP was the rs2066865, located in the 3′UTR region of the gene and known to influence both fibrinogen γ′ levels and VT risk [15], [23], [24]. The *F11* hit SNP was the rs10029715. According to the SNAP software [25] based on HapMap 3 (release 2), this SNP is in modest linkage disequilibrium (LD) with two *F11* SNPs recently found to independently affect VT risk [20], [26], rs2036914 and rs2289252 (r^2^ = 0.094, D′ = +0.66, and r^2^ = 0.076; D′ = +0.75, respectively). However, these two SNPs were not available in our genotyping array. Several other *F11* SNPs showed suggestive statistical associations with VT at p<10^−4^ (Table S1) and their haplotype analysis showed that the *F11* association signal was driven by two common yin/yang haplotypes (Table 2). We then used the HapMap data to infer the *F11* haplotypic structure derived from the two rs2036914 and rs2289252 SNPs and those found associated with VT in our sample. It is interesting to note that the yin-yang pattern described above is still present when the rs2036914 and rs2289252 are included in the analysis (Table S2).

**Table 1:** Stage I - Minor allele frequencies distribution of SNPs demonstrating association with VT at p_EIGENSTRAT_<2.0 10^−8^ in a GWAS sample of 1,542 VT cases and 1,110 controls.

CHR	Position	Gene	SNP	Alleles(1)	Cases	Controls	P(2)	P(3)
1	167401751	*NME7*	rs16861990	C/A	0.134	0.058	5.53×10^−20^	2.75×10^−15^
1	167695568	*SLC19A2*	rs1208134	C/T	0.133	0.056	4.06×10^−21^	3.29×10^−16^
1	167758179	*F5*	rs2420371	G/A	0.151	0.066	3.24×10^−23^	8.44×10^−19^
4	155744726	*FGG*	rs2066865	A/G	0.280	0.209	3.44×10^−9^	1.17×10^−10^
4	155720638	*FGA*	rs6825454	C/T	0.299	0.228	1.39×10^−8^	1.32×10^−9^
4	187459594	*F11*	rs10029715	C/T	0.115	0.172	1.09×10^−9^	3.20×10^−9^
9	135126961	*ABO*	rs2073828	A/G	0.321	0.406	1.91×10^−10^	3.57×10^−9^
9	135129086	*ABO*	rs657152	C/A	0.494	0.383	5.55×10^−20^	1.10×10^−18^
9	135138468	*ABO*	rs500498	T/C	0.332	0.432	3.54×10^−14^	1.03×10^−12^
9	135139050	*ABO*	rs505922	C/T	0.489	0.350	1.53×10^−25^	1.06×10^−23^
9	135139543	*ABO*	rs630014	A/G	0.381	0.485	9.26×10^−15^	4.40×10^−14^
9	135144688	*ABO*	rs495828	T/G	0.357	0.264	8.82×10^−13^	1.78×10^−14^

(1) Common/minor alleles.

(2) P-value of the Cochran-Armitage Trend test.

(3) Association test p-value corrected for principal components (EIGENSTRAT program).

**Table 2:** Haplotype association analysis of *F11* hit SNPs with VT risk in a sample of 1,542 VT cases and 1,110 controls.

Polymorphisms				Haplotype Frequencies	
rs925451	rs10029715	rs1008728	rs13133050	Controls	Cases
				n = 1110	n = 1542
A	T	T	C	0.338	0.403
A	T	C	A	0.033	0.027
A	C	C	C	0.016	0.011
G	T	T	C	0.257	0.248
G	T	C	A	0.196	0.201
G	C	C	C	0.043	0.041
G	C	C	A	0.106	0.051

*F11* haplotypes were more strongly associated with VT (p = 1.05 10^−12^) than single SNP alone (best p-value = 1.09 10^−9^) and the association was likely due to two common haplotypes, ATTC and GCCA, differing at all studied sites (“yin-yang” haplotypes), the former being associated with increased risk of VT, the latter with decreased risk. Compared to the GTTC haplotype, the ATTC haplotype was associated with an increased risk of VT (OR = 1.218 [1.048–1.416], p = 0.0099 while the GCCA haplotype was associated with a decreased risk of the disease (OR = 0.493 [0.391–0.623], p = 3.39 10^−9^).

#### Stage II

**Figure 4:**
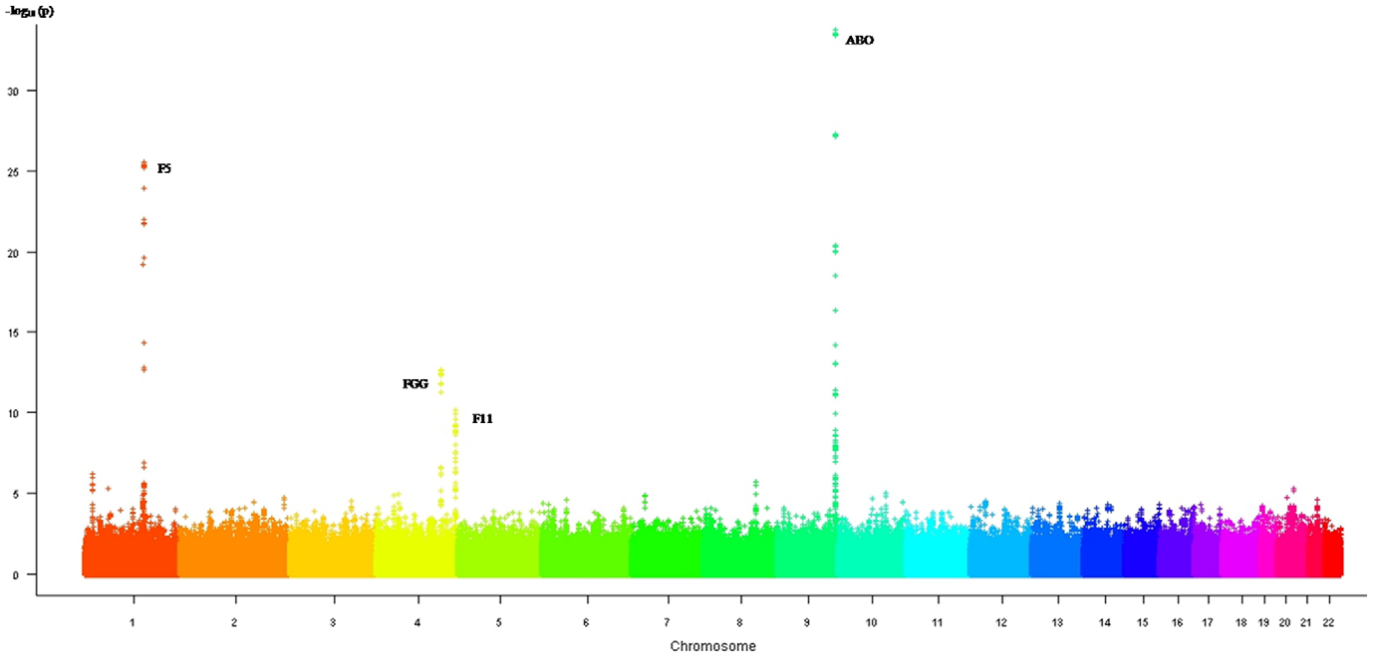
Manhattan plot of the association results from the combined analysis of two imputed GWAS data sets for 2,475,305 SNPs.

**Figure 5:**
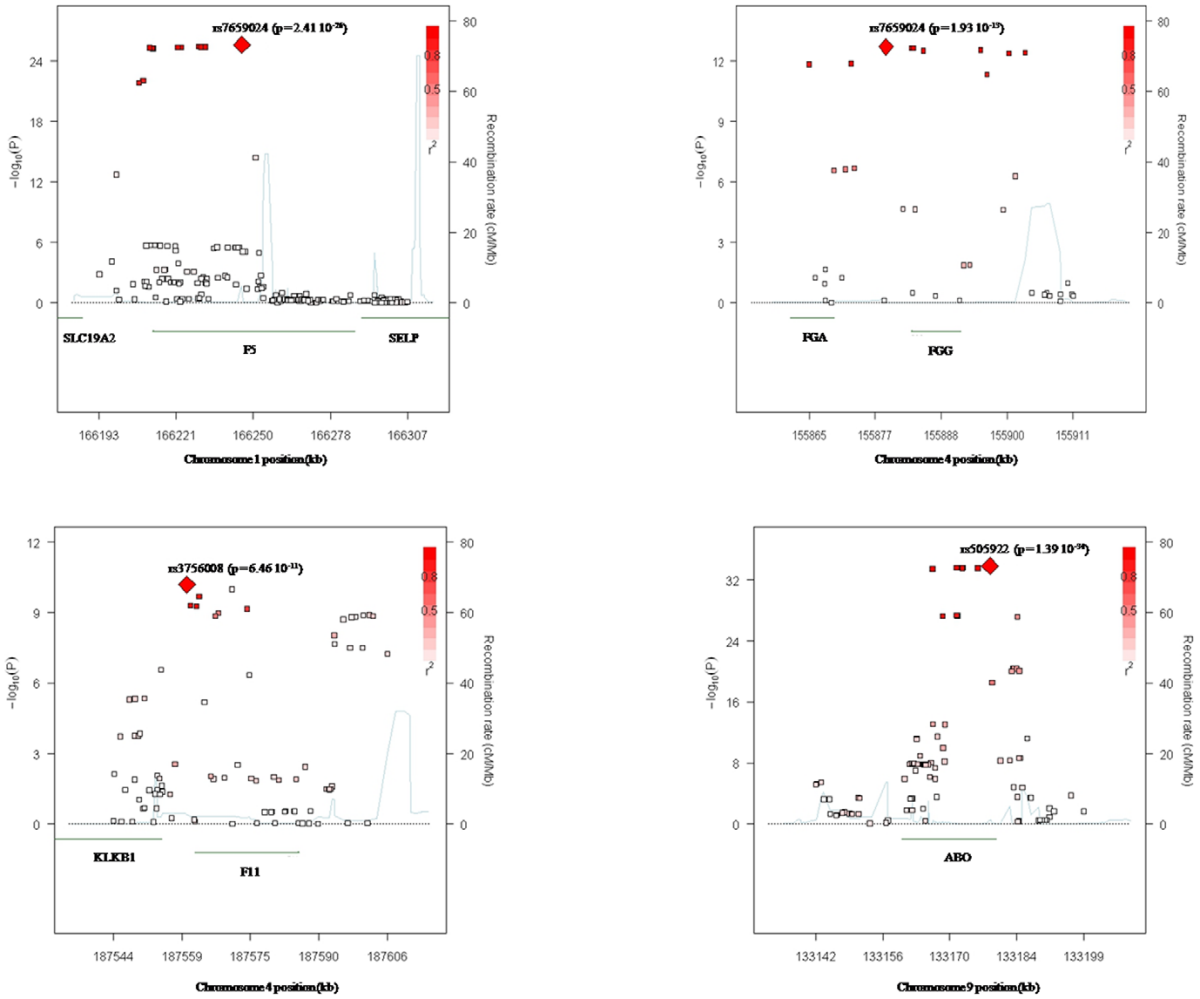
Regional association plots at the four genome-wide significant loci using imputed SNPs. Four genome-wide significant loci were *F5* (top left), *FGG* (top right), *F11* (bottom left) and *ABO* (bottom right). These plots were drawn from the SNAP software [25].

The results of the new GWAS were combined to those obtained on the previous *in silico* GWAS [15] through a meta-analysis totaling 1,961 VT cases and 2,238 controls. Using either the 253,355 genotyped SNPs common to both GWAS studies or 2,475,305 observed or imputed SNPs, no novel association was detected (Figure 4). In the imputation analysis, only 99 SNPs reached genome-wide significance and they were all located within the *ABO*, *F5*, *FGG*, or *F11* loci (Table S3). Detailed regional association plots for these four loci are shown in Figure 5.

### Candidate Gene association analysis

We then further explored the association results obtained in the discovery GWAS by focusing on SNPs located within candidate genes as it is now well-admitted that genuine association may be hidden in the heap of non genome-wide significant associations. Forty-nine genes were selected as candidates because they were either known to participate to the coagulation/fibrinolysis cascade, were already shown to be associated with VT , or had recently been identified through GWAS as modulating the variability of quantitative traits known/hypothesized to be associated with VT risk (Table S1).

In addition to the genome-wide significant loci discussed above, four candidate loci were found to harbor SNPs showing suggesting evidence of association with VT at p<10^−3^ (Table S1). These SNPs were rs169715 and rs2228220 in *HIVEP1*, rs6060278 and rs6088735 in *PROCR*, rs4981021 in *STAB2* and rs8074026 in *SERPINF2*.

The two *HIVEP1* SNPs, rs169715 and rs2228220, were in modest LD with each other (r^2^ = 0.09, D′ = +0.45). Their haplotype analysis suggested that the observed effects would be additive (Table 3), the rs169715-G allele being associated with an adjusted OR of 1.57 [1.17–2.09] (p = 2.60 10^−3^) and the rs2228220-G allele with an OR of 1.35 [1.10–1.66] (p = 3.98 10^−3^). In addition, this haplotype analysis suggested that the effect on VT risk of the previously identified *HIVEP1* rs169713 [17] was due to its LD with the rs169713 (r^2^ = 0.01, D′ = 1) and rs2228220 (r^2^ = 0.06, D′ = −0.50) *HIVEP1* hit SNPs (Table 3).

**Table 3:** Haplotype analysis of *HIVEP1* haplotypes derived from rs169713, rs169715 and rs2228220 in a sample of 1,542 cases and 1,110 controls.

Polymorphisms			Haplotype Frequencies	
rs169713	rs169715	rs2228220	Controls	Cases
			n = 1,110	n = 1,542
C	A	A	0.198	0.206
C	A	G	0.042	0.054
T	A	A	0.698	0.643
T	A	G	0.026	0.037
T	G	A	0.018	0.027
T	G	G	0.017	0.031

*HIVEP1* haplotypes were strongly associated with VT risk (χ^2^ = 30.22 with 5df, p = 1.33 10^−5^).

All haplotypes carrying the rs169715-G or the rs2228220-G alleles tended to be more frequent in cases than in controls, suggesting that both alleles could act additively to influence VT risk. This hypothesis were then tested and was not rejected (χ^2^ = 0.75 with 2 df, p = 0.686). After adjusting for rs2228220, the OR associated with the rs169715-G allele was 1.57 [1.17–2.09] (p = 2.60 10^−3^) and the OR associated with rs2228220-G adjusted for rs169715 was 1.35 [1.10–1.66] (p = 3.98 10^−3^). After adjusting for these two SNPs, the rs169713-C allele was not significant (OR = 1.11 [0.97–1.26], p = 0.129).

The two *PROCR* SNPs, rs6060278 and rs6088735, were in complete association (r^2^ = 1) and were also in strong negative LD (r^2^ = 0.03, D′ = −1) with the rs867186 variant, also known as Ser219Gly. The role of the latter in VT risk is still a matter of debate [15] and its association with VT was borderline in our discovery GWAS (Table S1). Nevertheless, a haplotype analysis of the rs6088735 and rs867186 suggested that both SNPs could act additively on the risk of disease (Table 4). When adjusted for rs6088735, the rs867186-G allele was associated with an OR for VT of 1.33 [1.11–1.60] (p = 2.34 10^−3^) whereas , adjusted for rs867816, the rs6088735-T allele was associated with an OR of 1.35 [1.19–1.54] (p = 5.47 10^−6^) (Table 4). Interestingly, according to the SNAP database [25], the rs6088735 is in complete association (r^2^ = 1) with the *EDEM2* rs6120849 that was recently found associated with protein C levels in the ARIC study [27]. The rs6088735-T allele associated with increased risk of VT corresponds to the rs6120849-T allele that was associated with decreased protein C levels, an observation consistent with the known association of decreased PROC levels and VT risk [28].

**Table 4:** Haplotype analysis of *PROCR* haplotypes derived from rs6088735 and rs867186 in a sample of 1,542 cases and 1,110 controls.

Polymorphisms		Haplotype Frequencies	
rs6088735	rs867186	Controls	Cases
		n = 1,110	n = 1,542
C	A	0.673	0.603
C	G	0.095	0.115
T	A	0.232	0.282

*PROCR* haplotypes were strongly associated with VT risk (χ^2^ = 26.51 with 2 df, p = 1.75 10^−6^). Compared to the most frequent CA haplotype, the CG and TA haplotypes were associated with an increased OR for VT of 1.33 [1.11–1.60] (p = 2.34 10^−3^) and 1.35 [1.19–1.54] (p = 5.47 10^−6^).

The *STAB2* rs4981021-T allele was associated with increased risk of VT 1.29 [1.14–1.46] (p = 3.17 10^−4^) but this association did not reach significance (OR = 1.10, p = 0.251) in the “in silico GWAS”. Conversely, the most significant *STAB2* SNP in the latter study was the rs1593812 already discussed in [29]. Haplotype analysis of these two SNPs suggested that they define at least one common at-risk haplotype for VT in both GWAS datasets (OR = 2.18 [1.66–2.87] (p = 2.16 10^−8^) (Table 5). Note that the rs4981021-T is a good proxy (r^2^ = 0.88) for the rs12229292-T not typed in our GWAS array but recently found associated with increased Factor VIII levels [30].

**Table 5:** Haplotype analysis of *STAB2* haplotypes derived from rs1593812 and rs4981021 in two GWAS data sets.

Polymorphisms		Haplotype Frequencies			
		“in silico GWAS”		Discovery GWAS	
rs1593812	rs4981021	Controls	Cases	Control	Cases
		n = 1,228	n = 419	n = 1,110	n = 1,542
A	C	0.629	0.573	0.646	0.587
A	T	0.248	0.240	0.219	0.246
G	C	0.091	0.114	0.104	0.111
G	T	0.032	0.073	0.031	0.056

Compared to the most frequent AC haplotype, the GT haplotype was associated with an increased risk of 2.43 [1.60–3.71] (p = 3.0 10^−5^) and 2.01 [1.40–2.88] (p = 1.48 10^−4^) in the “In silico” and discovery GWAS respectively. The combined Mantel-Haenszel OR associated with the GT haplotype compared to the AC haplotype was then 2.18 [1.66–2.87] (p = 2.16 10^−8^).

The association observed at *SERPINF2* was novel, the rs8074026-T allele being more frequent in MARTHA cases than in 3C healthy controls (0.29 vs 0.24, p = 6.87 10^−4^). This SNP was not typed in our previous GWAS in patients with early age of onset of VT but a similar trend was observed using imputation data (0.22 vs 0.19, p = 0.289). This SNP was therefore further explored in the FARIVE study, but the association was not confirmed as the rs8074026-T allele tended, conversely, to be less frequent in cases than in controls (0.23 vs 0.25, p = 0.520). This polymorphism was then not further studied.

### Genetic variance analysis

The next step of our analysis consisted in getting an overall estimate of the genetic variance (h^2^) of VT as well as an estimate of the contribution of the main susceptibility loci discussed above. The application of the GCTA software [31] to the discovery GWAS led to an estimate of 0.357±0.049. This estimate was obtained using an assumed prevalence of 0.001 for the disease according to [1]. This estimate was strongly dependent on the value of the assumed prevalence (Table 6). When the GCTA analysis was applied to the first GWAS data set with an assumed prevalence of 0.001, the genetic variance estimate was 0.223 with a larger standard error, 0.108, an estimate that was nevertheless consistent with that observed in the discovery GWAS.

**Table 6:** Relative contribution of each chromosome on the total genetic variance of VT according to the assumed prevalence of the disease.

	prevalence			
	0.001	0.005	0.01	0.05
Total Genetic	0.357	0.480	0.561	0.860
Variance ± SE	±0.047	±0.067	±0.078	±0.120
chromosome				
1	0.016	0.033	0.038	0.036
2	0.015	0.020	0.023	0.036
3	0.019	0.026	0.031	0.045
4	0.041	0.052	0.060	0.094
5	0.013	0.017	0.020	0.029
6	0.006	0.012	0.014	0.016
7	0.011	0.012	0.014	0.027
8	0.004	0.021	0.025	0.010
9	0.039	0.049	0.058	0.089
10	0.040	0.042	0.049	0.094
11	0.007	0.000	0.002	0.013
12	0.020	0.018	0.022	0.047
13	0.008	0.009	0.010	0.019
14	0.002	0.017	0.019	0.003
15	0.017	0.021	0.024	0.039
16	0.007	0.010	0.012	0.016
17	0.006	0.004	0.005	0.015
18	0.008	0.008	0.010	0.017
19	0.001	0.017	0.020	0.037
20	0.069(a)	0.081	0.094	0.159
21	0.008	0.009	0.011	0.019
22	0.000	0.000	0.000	0.000

Estimates were obtained from the discovery GWAS data and adjusted for gender and principal components.

(a) When chromosome 20 SNP data were split into two parts, one including 6,769 SNPs on the shortest 20p arm and the other 7,170 SNPs on the longest 20q arm, their relative contribution on the genetic variance were 0.056±0.013 and 0.013±0.007.

**Figure 6:**
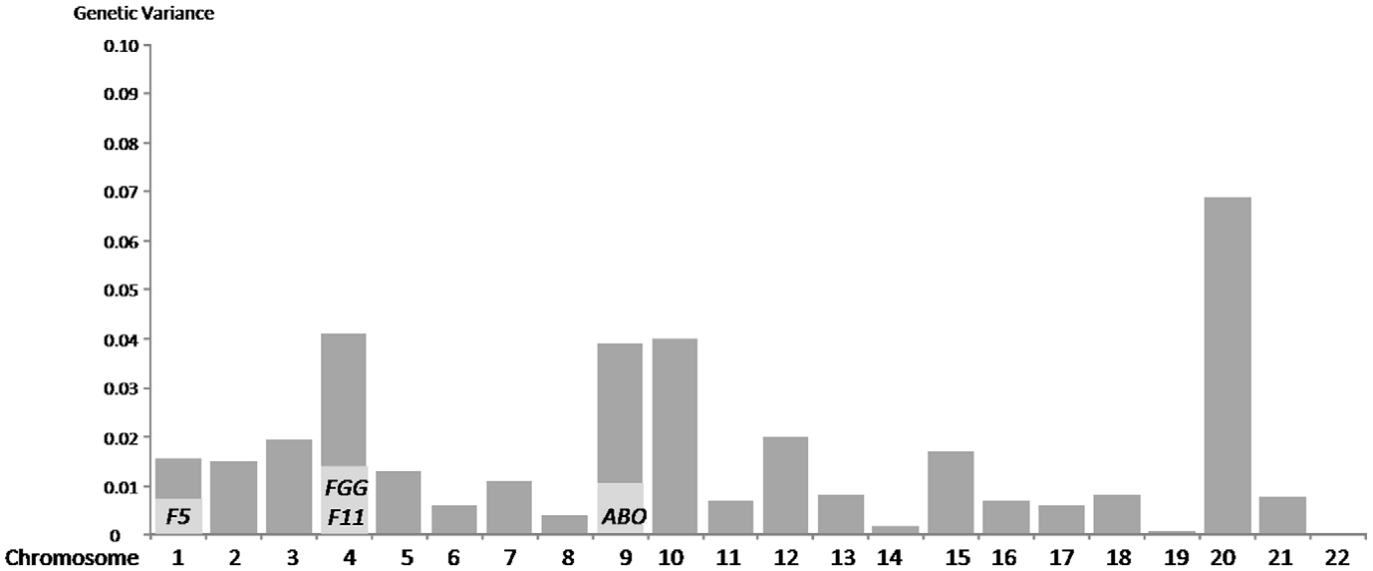
Distribution of VT genetic variance across chromosomes. In light grey is shown the relative contribution of specific loci.

The relative contribution of each chromosome is summarized in Figure 6 for a prevalence of 0.001. While *F5* and *ABO* explained ∼1%, each, of the genetic variance, and the *FGG* and *F11* together only 1.2%, we note that the chromosome most contributing to the estimate (6.9%±1.2) was chromosome 20. This observation hold whatever the assumed value for the prevalence of VT (Table 6). We then turned back to the original GWAS results and focused on chromosome 20 SNPs. Only eleven chromosome 20 SNPs corresponding to four different loci *RSPO4*, *C20orf23/SNRPB2*, *MYLK2* and *PREX1*, showed association at p<10^−4^ with VT (Table 7). We further investigated whether these four loci, in addition to the *PROCR* locus mentioned above that was also located on chromosome 20, could substantially contribute to explain the genetic variance contribution of chromosome 20. After discarding the genetic influence of these five loci, the remaining estimate associated with chromosome 20 SNPs was 5.5%±1.2. Further analyses also suggested that ∼80% of the chromosome 20's contribution came from SNPs located in its 20p arm (Table 6).

**Table 7:** Minor allele frequencies distribution of chromosome 20 SNPs demonstrating association with VT at p_EIGENSTRAT_<1.00×10^−4^ in a GWAS sample of 1,542 VT cases and 1,110 controls.

CHR	Position	Gene	SNP	Alleles(1)	Cases	Controls	P(2)
20	960026	*RSPO4*	rs11696364	C/A	0.059	0.102	1.53×10^−6^
20	16273269	*C20orf23*	rs4814475	G/A	0.181	0.228	3.98×10^−5^
20	16312593	*C20orf23*	rs6034465	C/T	0.158	0.204	6.73×10^−6^
20	16574082	*C20orf23*	rs964216	C/T	0.116	0.084	4.55×10^−5^
20	16575913	*C20orf23*	rs13038362	A/C	0.114	0.084	7.41×10^−5^
20	16580409	*SNRPB2*	rs6135823	T/C	0.115	0.083	5.38×10^−5^
20	29886239	*MYLK2*	rs17340555	T/C	0.110	0.084	4.53×10^−5^
20	46604745	*PREX1*	rs1883888	G/A	0.350	0.286	1.11×10^−5^
20	46613060	*PREX1*	rs878198	A/C	0.354	0.292	3.67×10^−5^
20	46622891	*PREX1*	rs4810820	T/C	0.364	0.306	9.95×10^−5^
20	46624742	*PREX1*	rs6012481	C/T	0.345	0.287	2.71×10^−5^

(1) Common/minor alleles.

(2) Association test p-value corrected for principal components (EIGENSTRAT program).

## Discussion

In this work we reported the results of a second GWAS on VT that, when added to the previous one, gathered a total sample of 1,961 cases and 2,338 controls, all of French origin. With such a sample size, our study had a power of 80% at the genome-wide significance level of ∼2×10^−8^ to detect the allelic effect of any SNP associated with an OR of 1.40 provided that its minor allele frequency is greater than 0.20 [32]. These values perfectly matched those observed at the four loci (*F5*, *FGG*, *F11* and *ABO*) that reached genome-wide significant in this report (Table S3), these four loci being now well-established susceptibility genes to VT [15], [16], [26]. The contribution of *F5* and *ABO* in VT susceptibility has been extensively discussed and the functional role of *FGG* rs2066865 has already been established [3], [23], [24]. Conversely, the identification of the functional *F11* variant(s) hypothesized to be tagged by the ying/yang haplotype structure discussed above deserves additional work.

In order to achieve a 80% power for detecting ORs of magnitude 1.30, 1.25 and 1.20, the statistical stringency would have to be lessened to 3×10^−5^, 6×10^−4^, and 9×10^−2^, respectively, at the risk of increasing false positive rates. In an attempt to increase the power of our analysis while limiting for false positives, we therefore focused on all SNPs located within ∼fifty candidate genes and demonstrating suggestive statistical evidence (p<10^−4^) for association with VT using raw genotype data. Suggestive associations were observed for the *HIVEP1*, *PROCR*, *STAB2* and *SERPINF2* loci (Table S1). Associations with VT have already been reported for the first three genes [15], [17], [29] and disentangling their exact genetic contribution to VT susceptibility would warrant additional extensive works. The latest suggestive association was observed for the *SERPINF2* rs8074026. *SERPINF2* is an obvious candidate for VT as it codes for a serpine protease inhibitor that acts as a inhibitor of plasmin. However, no trend for association was observed in the replication study.

Following the findings of a GWAS on aPTT levels [33], a candidate biomarker for VT, we have recently suggested that the *KNG1* Ile581Thr variant (rs710446) could also be a risk factor for VT using data from our discovery GWAS and the FARIVE studies. This variant only reached a significance of p = 1.17 10^−3^ in the discovery GWAS (Table S1) highlighting the need for exploring in more details the list of less significant p-values, in particular by use of external information on candidate quantitative risk factors. Two other SNPs have recently been suggested to influence VT-risk, *STXBP5* rs1039084 and *VWF* rs1063856 [34]. These were not available in the “in silico” GWAS, but using QC imputed data in the whole set of 1,961 cases and 2,338 controls, the rare allele of the *VWF* rs1063856 was marginally associated with the risk of VT (OR = 1.10 [1.00–1.21], p = 0.042) , an association consistent with that previously reported [34]. Conversely, we did not observe any trend of association for the *STXBP5* rs1039084 rare allele (OR = 0.97 [0.89–1.06]; p = 0.55), even if this OR was of similar amplitude with that observed in the MEGA study (OR = 0.91 [0.86–0.97]) [34]. These two associations were previously observed in a meta-analysis of studies gathering about 5,000 cases and 5,000 controls, underlying the low power of our study to detect modest genetic effect as already discussed above. Large GWAS samples gathering at least ∼20,000 patients would be required in order to detect genome-wide significant ORs of ∼1.10 and, for the moment, we are far from reaching such sample size by contrast to international consortia on coronary artery disease [35] . Another limitation of this work could be related to the selection of the GWAS subjects. Controls were part of a national GWAS sample of French healthy individuals that were not matched to VT cases, in particular for gender and sex. Nevertheless, all known or suspected VT-associated loci were identified in our work suggesting a rather modest influence of imperfect matching between cases and controls. Conversely, VT patients homozygous for the FV Leiden or FII 20210A mutation or with anti-thrombin, protein C or protein S deficiencies were not included in this work. It is very unlikely that the selection on FV Leiden homozygosity had affected our results as the *F5* gene is among the four loci that reached genome-wide significance in our study. Note that the FII 20210 mutation (rs1799963) was not available in the imputed reference datasets. However, one cannot exclude that the other exclusion criteria may have affect our power to identify novel VT-associated variants, in particular through a modulation of anti-thrombiin, protein C or protein S levels. It is nevertheless worthy of note that the *PROCR* locus that was found influencing the most protein C levels in the ARIC GWAS [27], was among the top 8 most significant VT-associated loci in our GWAS.

The second original aspect of our work is the application of a novel statistical methodology to get an estimate of the genetic variance of VT. This approach requires several assumptions including a fixed value for the disease prevalence, additive genetic effects and the existence of an underlying liability characterized by a threshold above which the disease status is called. Using the latest known estimate of the VT prevalence [1], we showed that the genetic variance could be ∼35%, an estimate slightly lower than those obtained from families studies [6], [7]. While the four main VT-linked loci, *FV*, *ABO*, *FGG* and *F11*, altogether contributed to about ∼3% of the total genetic variance it was striking to observe that chromosome 20 was the chromosome contributing the most to the total genetic variance with about ∼7% of the total genetic variance. Further analyses including chromosome-wide haplotype and homozygosity mapping analyses are ongoing to further investigate the chromosome 20 genetic architecture in relation to VT risk.

In conclusion, this work provided new information about the genetic susceptibility to VT and strongly suggested that chromosome 20 genes warrant specific attentions. It generated a wealth of valuable genetic information to those showing interest in disentangling the genetic architecture of VT.

## Materials and Methods

### Ethics Statement

Each individual study was approved by its institutional ethics committee and informed written consent was obtained in accordance with the Declaration of Helsinki. All subjects were of European origin. All subjects were of European origin.

Ethics approval were obtained :

- for MARTHA, from the “Departement santé de la direction générale de la recherche et de l'innovation du ministère” (Projects DC: 2008-880 & 09.576).
- for FARIVE, from the “Comité consultatif de protection des personnes dans la recherche biomedicale” (Project n° 2002-034)
- for the 3C study, from the institutional ethics committees of the Kremlin-Bicetre Hospital.

### Studies

#### Stage I - Discovery GWAS

MARTHA patients (n = 1,592) are unrelated VT patients, mainly of French origin, consecutively recruited at the Thrombophilia center of La Timone hospital (Marseille, France) between January 1994 and October 2005. All patients had a history of a first VT event documented by venography, Doppler ultrasound, angiography and/or ventilation/perfusion lung scan. They were all free of any chronic conditions and free of any well characterized genetic risk factors including anti-thrombin, protein C or protein S deficiency, homozygosity for FV Leiden or FII 20210A, and lupus anticoagulant. A more detailed description of these patients can be found in [21]. These VT patients were compared to healthy individuals from the 3C study.

The 3C Study is a population-based, prospective (4-years follow-up) study, initially set-up to investigate the relationship between vascular factors and dementia. It has been carried out in three French cities: Bordeaux (southwest France), Montpellier (southeast France) and Dijon (central eastern France). A sample of non-institutionalised subjects aged over 65 was randomly selected from the electoral rolls of each city. Between January 1999 and March 2001, 9,686 subjects meeting the inclusion criteria agreed to participate. Following recruitment, 392 subjects withdrew from the study. Thus, 9,294 subjects were finally included in the study (2,104 in Bordeaux, 4,931 in Dijon and 2,259 in Montpellier). At the baseline clinical examination, blood samples were obtained from 8,707 individuals. For the present study, a random sample of 1,140 subjects free of any chronic diseases was selected to serve as controls.

#### Stage II - *In silico* GWAS study

In a previously published GWAS on VT [15], 419 early age of onset (<50 years) VT cases were compared to 1,228 healthy controls at 291,872 SNPs. Cases were patients from four different French medical centers (Grenoble, Marseille, Montpellier, Paris) selected according to the same criteria as the MARTHA patients, except with the restriction on age of onset. Controls were French subjects selected from the SUVIMAX population [36].

#### Stage III - Replication studies

For the replication of the GWAS findings, the FARIVE study [15], a multicenter case-control study for first episode of VT composed of 607 cases and 607 healthy individuals, all of French origin, was used.

### Genotyping and Quality control

#### Stage I - Discovery GWAS

A subsample of 1011 VT patients were typed with the Illumina Human 610-Quad Beadchip while the remaining 586 VT patients were typed with the Illumina Human660W-Quad Beadchip. Individuals from the 3C study were also typed with Illumina Human 610-Quad Beadchip. A set of 551,141 SNPs including 537,883 autosomal SNPs and 13,258 sex-linked SNPs was common to the three samples.

Individuals with genotyping success lower than 95% (n = 18) were excluded from the analyses as were individuals demonstrating close relatedness (n = 67). This latter was assessed by pairwise clustering of identity by state distance (IBS) and multi-dimensional scaling (MDS) using the PLINK software [37]. The Eigenstrat program [38] was further used to detect individuals of non-European ancestry. SNPs showing significant (p<10^−5^) deviation from Hardy-Weinberg Equilibrium (HWE) in controls, with minor allele frequency (MAF) less than 1% in the combined cases/controls samples or genotyping call rate <99% were filtered out. This lead to the final analysis of 481,002 autosomal and 10,256 sex-linked SNPs in a sample of 1,542 VT patients and 1110 healthy individuals.

#### Stage II - *In silico* GWAS study

Individuals participating in this previous GWAS were genotyped for 317,139 SNPs using the Illumina Sentrix HumanHap300 Beadchip among which 291,872 satisfied the quality control criteria previously described [15]. Individuals of non European ancestry had been also excluded from this analysis [15].

#### Stage III - Replication studies

In FARIVE, the rs8074026 was genotyped by allele-specific PCR (also referred to as ARMS i.e amplification refractory mutation system) with success rate of 97.5%.

### Statistical Analysis

#### Genome-wide association study

Genome-wide association analysis of autosomal SNPs was conducted using the Eigenstrat program that correct for any uncontrolled population stratification [38]. The genomic control (GC) inflation factor was also computed according to the median test statistic [39]. X-linked SNPs association was tested using the PLINK software [37] while adjusting for first four principal components.

#### Haplotype analysis

To handle the linkage disequilibrium (LD) between SNPs of interest at specific loci, haplotype analysis was performed by use of the THESIAS program [40].

#### Imputation

In both GWAS datasets, imputation of 2,557,252 autosomal SNPs was conducted using the MACH (v1.0.16a) software (http://www.sph.umich.edu/csg/abecasis/mach/) according to the CEU HapMap 2 release 21 (build 35) reference dataset. A logistic regression analysis was then conducted to evaluate the association of each SNP with VT risk in an additive genetic model, in which allele dosage (0 to 2 copies of the minor allele) of imputed SNPs was analyzed. Analyses were adjusted for the first four principal components and were performed using the mach2dat (v 1.08.18) software (http://genome.sph.umich.edu/wki/Minimac).

#### Meta-Analysis

All SNPs with acceptable imputation quality (r2≥0.3) in both imputed GWAS datasets were entered into a meta-analysis, leading to 2,475,305 SNPs left for statistical association analysis. For the meta-analysis, a fixed-effect model relying on the inverse-variance weighting was used as implemented in the METAL software (http://www.sph.umich.edu/csg/abecasis/metal). Homogeneity of associations across the two GWAS studies was tested using the Mantel-Haenszel method [41].

For all these GWAS analyses, a statistical threshold of 2.0×10^−8^ was used to declare genome-wide significance. This value corresponds to the family-wise error rate of 0.05 corrected for the number of studied SNPs (2,475,305) according to Bonferroni correction.

#### Replication

Association of SNPs tested for replication with VT was assessed by use of the Cochran-Armitage trend test [42]. Logistic regression analysis was further used to estimate genetic effects, expressed in terms of Odds Ratio (OR), adjusted for age, gender, FV leiden and ABO blood group.

#### Genetic Variance Estimation

The recently proposed GCTA methodology was used to investigate the genetic variance of VT [31], [43]. Briefly, this method consists in estimating the genetic relationship between unrelated individuals from genome-wide SNPs information and in incorporating it into a regression model to provide an estimate of the genetic variance of a given phenotype. For a binary phenotype such as VT, it assumes the existence of an underlying normally distributed liability variable, with individuals being affected if their liability exceeds a threshold which may depends on covariates such as gender. We computed the genetic relationships separately from all SNPs of a given chromosome and assessed the contribution of each chromosome on the genetic variance. A similar approach was applied to estimate the contribution of specific loci of using all SNPs within ±5 Mb of each locus. All analyses were adjusted for gender and principal components as indicated in the GCTA documentation [31].

## Supporting Information

Table S1 **Allele frequencies of candidate gene SNPs in the discovery GWAS sample of 1,542 VT cases and 1,110 controls.** ^(1)^ Common/minor alleles. ^(2)^Association test p-value corrected for principal components (EIGENSTRAT program). ^(3)^ P-value of the Cochran-Armitage Trend test corrected for the genomic control factor. Genes were selected as candidates for VT because either: - (A): they belong to the coagulation cascade (Blood 2000; 95:1517–1532;Blood 2008; 112: 19–27). - (B): or they belong to the fibrinolytic cascade (Blood 2000; 95:1517–1532; Semin Thromb Hemost. 2009;35:468–77). - (C): or they harbours SNPs that have been associated with VT risk (Blood 2010; 115:4644–4650; JAMA 2008; 299:1306–1314; Am J Hum Genet 2010; 86:592–595; J Thromb Haemost 2010; 8:2671–2679). - (D): or they mapped loci found through recent GWAS associated with quantitative biomarkers of VT such as D1: vWF & FVIII (Circulation 2010; 121:1382–1392). D2: Platelet volume (Am J Hum Genet 2009; 84:66–71). D3: Protein C levels (Blood 2010; 116:5032–5036). D4: aPTT (Am J Hum Genet 2010; 86:626–631). D5: PAI-1 levels (Blood 2010; 116:2160–2163).
(DOC)

Table S2 **Haplotype structure derived from VT-associated** ***F11*** **SNPs in the HapMap database.** Haplotype frequencies were estimated using the Haploview software from the HapMap 3 (release 2) data. SNPs identified in the LETS study (Li Y et al. J Thromb Haemost 2009;7:1802–1808) are shown in bold, others were those identified in the current MARTHA project.
(DOC)

Table S3 **Stage II - Genome-wide significant (p<2.01 10^−8^) SNP imputed associations with VT in the combined discovery and** ***in silico*** **GWASes of 1,961 cases and 2,338 controls.** ^(1)^ Common/rare alleles. ^(2)^ Minor allele frequency. ^(3)^ Odds Ratio associated with the minor allele estimated from the Mach2dat imputation software, after adjusting for principal components. ^(4)^ Combined p-values computed using the inverse-variance model as implemented in METAL software. All shown imputed SNPs satisfied the imputation quality criteria (r2.hat>0.3).
(DOC)
