# Supplementary material for: Genetics of Venous Thrombosis: Insights from a New Genome Wide Association Study
Source: PLoS One. 2011 Sep 27;6(9):e25581. doi: 10.1371/journal.pone.0025581 (PMC3181335; doi:10.1371/journal.pone.0025581)
Supplement: Table S1 — Allele frequencies of candidate gene SNPs in the discovery GWAS sample of 1,542 VT cases and 1,110 controls. (1) Common/minor alleles. (2)Association test p-value corrected for principal components (EIGENSTRAT program). (3) P-value of the Cochran-Armitage Trend test corrected for the genomic control factor. Genes were selected as candidates for VT because either: - (A): they belong to the coagulation cascade (Blood 2000; 95:1517–1532;Blood 2008; 112: 19–27). - (B): or they belong to the fibrinolytic cascade (Blood 2000; 95:1517–1532; Semin Thromb Hemost. 2009;35:468–77). - (C): or they harbours SNPs that have been associated with VT risk (Blood 2010; 115:4644–4650; JAMA 2008; 299:1306–1314; Am J Hum Genet 2010; 86:592–595; J Thromb Haemost 2010; 8:2671–2679). - (D): or they mapped loci found through recent GWAS associated with quantitative biomarkers of VT such as D1: vWF & FVIII (Circulation 2010; 121:1382–1392). D2: Platelet volume (Am J Hum Genet 2009; 84:66–71). D3: Protein C levels (Blood 2010; 116:5032–5036). D4: aPTT (Am J Hum Genet 2010; 86:626–631). D5: PAI-1 levels (Blood 2010; 116:2160–2163). (DOC) [file pone.0025581.s001.doc]

**Table S1.** Allele frequencies of candidate gene SNPs in the discovery GWAS sample of 1,542 VT cases and 1,110 controls.

| CHR | Gene | Position | SNP | Alleles^(1)^ | Cases | Controls | P^(2)^ | P^(3)^ |
| --- | --- | --- | --- | --- | --- | --- | --- | --- |
| 1 | F3 ^(A)^ | 94776672 | rs762485 | A/C | 0.448 | 0.447 | 0.500 | 0.974 |
|  |  | 94776998 | rs762484 | T/C | 0.252 | 0.247 | 0.305 | 0.745 |
|  |  | 94777808 | rs696619 | G/A | 0.435 | 0.435 | 0.608 | 0.989 |
|  |  | 94796253 | rs12725867 | T/C | 0.146 | 0.151 | 0.341 | 0.685 |
|  |  | 94798009 | rs2762509 | T/C | 0.165 | 0.16 | 0.295 | 0.695 |
|  |  | 94798607 | rs17111713 | T/G | 0.089 | 0.084 | 0.289 | 0.644 |
|  |  | 94801032 | rs17365315 | G/A | 0.075 | 0.076 | 0.632 | 0.959 |
|  |  | 94824518 | rs1041042 | A/G | 0.174 | 0.173 | 0.309 | 0.956 |
|  |  | 94824855 | rs12758064 | T/G | 0.114 | 0.117 | 0.297 | 0.756 |
|  |  | 94825064 | rs2022309 | G/T | 0.317 | 0.313 | 0.98 | 0.77 |
|  |  | 94827528 | rs841680 | C/A | 0.212 | 0.219 | 0.691 | 0.653 |
|  |  | 94827720 | rs1772904 | C/T | 0.173 | 0.173 | 0.325 | 0.986 |
|  |  | 94840935 | rs1772895 | G/A | 0.425 | 0.435 | 0.675 | 0.575 |
|  |  | 94841509 | rs4847313 | G/A | 0.32 | 0.338 | 0.569 | 0.261 |
|  |  | 94842629 | rs7542900 | C/T | 0.205 | 0.194 | 0.365 | 0.436 |
|  |  | 94847043 | rs1245489 | A/G | 0.429 | 0.443 | 0.378 | 0.377 |
|  |  | 94854175 | rs17411335 | A/C | 0.258 | 0.273 | 0.262 | 0.306 |
|  |  | 94856424 | rs10874867 | C/T | 0.322 | 0.311 | 0.260 | 0.478 |
|  |  | 94857846 | rs10782983 | C/T | 0.394 | 0.38 | 0.177 | 0.393 |
|  |  | 94859366 | rs3814018 | C/T | 0.383 | 0.363 | 0.115 | 0.199 |
|  |  | 94860677 | rs7550601 | C/T | 0.159 | 0.169 | 0.29 | 0.403 |
|  |  | 94863265 | rs12738550 | G/A | 0.277 | 0.300 | 0.108 | 0.119 |
|  |  | 94865939 | rs12725602 | G/A | 0.458 | 0.471 | 0.559 | 0.447 |
|  |  | 94866477 | rs11576456 | G/A | 0.181 | 0.169 | 0.198 | 0.318 |
|  |  | 94872128 | rs2211318 | A/G | 0.433 | 0.436 | 0.976 | 0.859 |
|  |  | 94874053 | rs10874870 | G/A | 0.404 | 0.39 | 0.225 | 0.369 |
|  |  | 94875738 | rs4847320 | C/T | 0.169 | 0.173 | 0.569 | 0.747 |
|  |  | 94879842 | rs2996284 | T/C | 0.178 | 0.169 | 0.394 | 0.448 |
|  | SERPINC1^(A)^ | 172126805 | rs16846546 | T/C | 0.115 | 0.102 | 0.364 | 0.215 |
|  |  | 172132209 | rs1322780 | C/T | 0.386 | 0.361 | 0.825 | 0.116 |
|  |  | 172135578 | rs6691053 | C/T | 0.247 | 0.23 | 0.399 | 0.234 |
|  |  | 172136266 | rs2295957 | C/T | 0.132 | 0.128 | 0.789 | 0.683 |
|  |  | 172142024 | rs2227612 | T/G | 0.134 | 0.126 | 0.415 | 0.49 |
|  |  | 172158724 | rs1951626 | G/A | 0.346 | 0.337 | 0.699 | 0.532 |
|  | F13B ^(B)^ | 195272506 | rs1332669 | T/C | 0.098 | 0.091 | 0.936 | 0.524 |
|  |  | 195274102 | rs857021 | G/A | 0.097 | 0.091 | 0.974 | 0.539 |
|  |  | 195278259 | rs7518773 | A/G | 0.493 | 0.473 | 0.565 | 0.24 |
|  |  | 195278734 | rs10754210 | G/A | 0.344 | 0.317 | 0.291 | 0.089 |
|  |  | 195294229 | rs1759007 | G/A | 0.095 | 0.09 | 0.929 | 0.619 |
|  |  | 195297644 | rs6003 | T/C | 0.097 | 0.091 | 0.986 | 0.545 |
|  | C4BPB ^(C)^ | 205330452 | rs6690037 | A/G | 0.421 | 0.456 | 0.459 | 0.033 |
|  |  | 205336481 | rs3813948 | T/C | 0.084 | 0.081 | 0.407 | 0.709 |
|  | C4BPA ^(C)^ | 205345074 | rs11120218 | G/A | 0.174 | 0.155 | 0.062 | 0.572 |
|  |  | 205353810 | rs2808470 | C/T | 0.226 | 0.202 | 0.597 | 0.084 |
|  |  | 205356059 | rs2842704 | A/G | 0.173 | 0.167 | 0.207 | 0.642 |
|  |  | 205358728 | rs9943268 | G/T | 0.386 | 0.374 | 0.7 | 0.462 |
|  |  | 205371523 | rs4844573 | T/C | 0.348 | 0.352 | 0.224 | 0.822 |
|  |  | 205372211 | rs4571969 | C/T | 0.265 | 0.259 | 0.538 | 0.677 |
|  |  | 205372293 | rs4266889 | G/A | 0.341 | 0.349 | 0.222 | 0.633 |
|  |  | 205378104 | rs7524207 | T/C | 0.036 | 0.027 | 0.966 | 0.116 |
| 2 | GCKR ^(D3)^ | 27584444 | rs1260326 | C/T | 0.475 | 0.466 | 0.597 | 0.597 |
|  |  | 27584716 | rs3817588 | T/C | 0.159 | 0.188 | 0.211 | 0.017 |
|  |  | 27594741 | rs780094 | C/T | 0.475 | 0.459 | 0.895 | 0.342 |
|  |  | 27596658 | rs780092 | A/G | 0.137 | 0.162 | 0.174 | 0.031 |
|  |  | 27596927 | rs11681351 | G/A | 0.326 | 0.319 | 0.266 | 0.65 |
|  | PROC^(A)^ | 127894885 | rs2069916 | C/T | 0.33 | 0.329 | 0.64 | 0.942 |
|  |  | 127906525 | rs1568277 | C/T | 0.142 | 0.169 | 7.08 10^-3^ | 0.022 |
|  |  | 127907566 | rs4662730 | C/A | 0.322 | 0.338 | 0.092 | 0.286 |
|  |  | 127910398 | rs10928766 | G/A | 0.141 | 0.167 | 8.96 10^-3^ | 0.028 |
|  |  | 127911714 | rs6754772 | G/A | 0.481 | 0.478 | 0.711 | 0.833 |
|  |  | 127911857 | rs6754999 | G/A | 0.345 | 0.341 | 0.634 | 0.785 |
|  |  | 127911912 | rs6755028 | A/G | 0.377 | 0.356 | 0.193 | 0.187 |
|  |  | 127912868 | rs11683986 | C/T | 0.327 | 0.312 | 0.217 | 0.348 |
|  |  | 127912894 | rs6749002 | T/G | 0.483 | 0.475 | 0.509 | 0.661 |
|  | TFPI^(A)^ | 188032097 | rs13424790 | T/G | 0.331 | 0.32 | 0.773 | 0.465 |
|  |  | 188051742 | rs7586970 | T/C | 0.329 | 0.317 | 0.918 | 0.443 |
|  |  | 188071820 | rs8176441 | T/C | 0.02 | 0.02 | 1 | 0.965 |
|  |  | 188078477 | rs3755248 | T/C | 0.341 | 0.339 | 0.343 | 0.902 |
|  |  | 188096556 | rs12613071 | T/C | 0.229 | 0.24 | 0.367 | 0.444 |
|  |  | 188098357 | rs13035938 | A/G | 0.274 | 0.268 | 0.982 | 0.678 |
|  |  | 188099064 | rs2192825 | T/C | 0.401 | 0.398 | 0.365 | 0.875 |
|  |  | 188118596 | rs10179730 | A/G | 0.103 | 0.128 | 0.011 | 0.016 |
|  |  | 188122406 | rs10187622 | C/T | 0.143 | 0.175 | 0.011 | 6.56 10^-3^ |
|  |  | 188127835 | rs10153820 | G/A | 0.123 | 0.122 | 0.556 | 0.899 |
|  |  | 188143888 | rs6730608 | G/T | 0.446 | 0.417 | 0.024 | 0.075 |
|  |  | 188148997 | rs12471440 | G/A | 0.391 | 0.413 | 0.164 | 0.176 |
|  |  | 188158381 | rs11902917 | T/C | 0.118 | 0.118 | 0.453 | 0.951 |
|  |  | 188160769 | rs4268904 | C/T | 0.446 | 0.417 | 0.026 | 0.082 |
|  |  | 188169683 | rs6434225 | C/T | 0.392 | 0.413 | 0.177 | 0.186 |
|  |  | 188179462 | rs4263080 | C/T | 0.042 | 0.048 | 0.525 | 0.319 |
|  |  | 188179516 | rs4622677 | A/C | 0.163 | 0.17 | 0.258 | 0.595 |
|  |  | 188191178 | rs7561970 | A/G | 0.181 | 0.175 | 0.757 | 0.669 |
|  |  | 188200108 | rs1356868 | A/C | 0.154 | 0.159 | 0.89 | 0.658 |
|  |  | 188216256 | rs1464339 | G/A | 0.154 | 0.158 | 0.979 | 0.736 |
|  |  | 188217305 | rs10198468 | G/A | 0.103 | 0.106 | 0.958 | 0.792 |
| 3 | ARHGEF3 ^(D2)^ | 56710220 | rs6773931 | C/A | 0.399 | 0.4 | 0.792 | 0.952 |
|  |  | 56715141 | rs6445815 | A/G | 0.39 | 0.392 | 0.843 | 0.905 |
|  |  | 56724942 | rs6773569 | T/C | 0.125 | 0.105 | 0.992 | 0.062 |
|  |  | 56734732 | rs4681767 | C/T | 0.401 | 0.398 | 0.989 | 0.86 |
|  |  | 56734840 | rs6787106 | A/G | 0.158 | 0.151 | 0.26 | 0.569 |
|  |  | 56738268 | rs1009119 | T/C | 0.155 | 0.151 | 0.192 | 0.724 |
|  |  | 56744756 | rs9311618 | T/C | 0.129 | 0.109 | 0.892 | 0.061 |
|  |  | 56744918 | rs9311619 | T/C | 0.13 | 0.11 | 0.883 | 0.064 |
|  |  | 56746291 | rs3772219 | A/C | 0.268 | 0.289 | 0.928 | 0.159 |
|  |  | 56751948 | rs4681794 | T/C | 0.272 | 0.289 | 0.853 | 0.248 |
|  |  | 56754051 | rs2046823 | G/A | 0.224 | 0.222 | 0.129 | 0.912 |
|  |  | 56758846 | rs3821413 | T/C | 0.387 | 0.379 | 0.721 | 0.61 |
|  |  | 56760596 | rs6792241 | C/T | 0.226 | 0.225 | 0.617 | 0.91 |
|  |  | 56763436 | rs1472038 | T/C | 0.488 | 0.474 | 0.182 | 0.387 |
|  |  | 56764783 | rs2171855 | A/G | 0.215 | 0.218 | 0.403 | 0.85 |
|  |  | 56771220 | rs9825368 | C/T | 0.102 | 0.108 | 0.814 | 0.531 |
|  |  | 56773025 | rs7635478 | G/A | 0.016 | 0.011 | 1 | 0.274 |
|  |  | 56780416 | rs9882898 | A/G | 0.14 | 0.14 | 0.348 | 0.977 |
|  |  | 56784668 | rs7646054 | G/A | 0.364 | 0.348 | 0.12 | 0.319 |
|  |  | 56797417 | rs7611544 | T/C | 0.485 | 0.497 | 0.058 | 0.263 |
|  |  | 56801831 | rs17216768 | T/C | 0.397 | 0.414 | 0.038 | 0.273 |
|  |  | 56804701 | rs12635549 | G/A | 0.186 | 0.158 | 0.029 | 0.024 |
|  |  | 56805127 | rs4681888 | T/C | 0.189 | 0.191 | 0.401 | 0.901 |
|  |  | 56805499 | rs7622820 | C/T | 0.024 | 0.018 | 0.513 | 0.187 |
|  |  | 56806588 | rs6803697 | G/A | 0.381 | 0.38 | 0.267 | 0.971 |
|  |  | 56809019 | rs1392702 | T/C | 0.475 | 0.48 | 0.236 | 0.797 |
|  |  | 56818068 | rs12638861 | T/G | 0.055 | 0.064 | 0.077 | 0.21 |
|  |  | 56821109 | rs2133884 | G/T | 0.468 | 0.468 | 0.233 | 0.989 |
|  |  | 56822871 | rs1566487 | A/G | 0.322 | 0.316 | 0.145 | 0.717 |
|  |  | 56824745 | rs1948722 | C/T | 0.036 | 0.035 | 0.209 | 0.791 |
|  |  | 56824789 | rs1354034 | C/T | 0.42 | 0.432 | 0.022 | 0.484 |
|  |  | 56827429 | rs17288929 | G/A | 0.13 | 0.129 | 0.219 | 0.962 |
|  |  | 56832473 | rs1344142 | C/T | 0.452 | 0.465 | 0.245 | 0.42 |
|  |  | 56832827 | rs11929318 | C/T | 0.127 | 0.14 | 0.228 | 0.277 |
|  |  | 56834464 | rs10866003 | C/T | 0.292 | 0.295 | 0.381 | 0.813 |
|  |  | 56838443 | rs1039379 | C/T | 0.189 | 0.187 | 0.537 | 0.868 |
|  |  | 56840792 | rs4681908 | C/T | 0.091 | 0.094 | 0.786 | 0.749 |
|  |  | 56841416 | rs7630830 | G/A | 0.456 | 0.446 | 0.441 | 0.541 |
|  |  | 56845574 | rs9881237 | G/A | 0.122 | 0.125 | 0.695 | 0.787 |
|  |  | 56849073 | rs7636889 | A/G | 0.244 | 0.258 | 0.413 | 0.33 |
|  |  | 56849416 | rs11717281 | C/T | 0.122 | 0.135 | 0.25 | 0.221 |
|  |  | 56852828 | rs10510789 | C/T | 0.162 | 0.15 | 0.771 | 0.323 |
|  |  | 56855484 | rs11130549 | T/C | 0.322 | 0.314 | 0.035 | 0.589 |
|  |  | 56866486 | rs6445829 | T/C | 0.439 | 0.46 | 0.063 | 0.221 |
|  |  | 56871425 | rs7630738 | A/G | 0.44 | 0.405 | 7.56 10^-3^ | 0.035 |
|  |  | 56877136 | rs13317313 | C/A | 0.12 | 0.109 | 0.327 | 0.311 |
|  |  | 56889105 | rs1500710 | A/C | 0.395 | 0.375 | 0.164 | 0.213 |
|  |  | 56889194 | rs1500711 | T/C | 0.447 | 0.472 | 0.148 | 0.143 |
|  |  | 56890759 | rs6445834 | C/T | 0.356 | 0.352 | 0.992 | 0.81 |
|  |  | 56899751 | rs11916112 | G/A | 0.224 | 0.247 | 0.092 | 0.105 |
|  |  | 56902063 | rs4681929 | G/A | 0.177 | 0.183 | 0.486 | 0.648 |
|  |  | 56904106 | rs874404 | C/T | 0.241 | 0.235 | 0.136 | 0.663 |
|  | PROS1^(A)^ | 95019980 | rs9681884 | G/A | 0.053 | 0.051 | 0.424 | 0.804 |
|  |  | 95028094 | rs9283589 | T/C | 0.03 | 0.022 | 0.129 | 0.134 |
|  |  | 95067163 | rs9290469 | C/T | 0.239 | 0.222 | 0.976 | 0.218 |
|  |  | 95129086 | rs8178610 | C/T | 0.415 | 0.401 | 0.136 | 0.373 |
|  |  | 95136682 | rs8178607 | G/A | 0.203 | 0.226 | 0.506 | 0.09 |
|  |  | 95164179 | rs8178591 | C/T | 0.203 | 0.227 | 0.483 | 0.081 |
|  |  | 95171201 | rs13062355 | A/G | 0.474 | 0.477 | 0.705 | 0.879 |
|  |  | 95176907 | rs7644769 | T/C | 0.418 | 0.403 | 0.128 | 0.369 |
|  | HRG^(D4)^ | 187864272 | rs9824398 | C/T | 0.429 | 0.414 | 0.483 | 0.347 |
|  |  | 187864410 | rs9879899 | T/C | 0.431 | 0.416 | 0.575 | 0.353 |
|  |  | 187866881 | rs11708008 | G/A | 0.053 | 0.049 | 0.901 | 0.565 |
|  |  | 187869993 | rs16860974 | T/G | 0.166 | 0.169 | 0.46 | 0.803 |
|  |  | 187870722 | rs2555515 | T/C | 0.367 | 0.394 | 0.441 | 0.102 |
|  |  | 187872253 | rs10770 | T/C | 0.14 | 0.126 | 0.338 | 0.196 |
|  |  | 187873321 | rs9898 | C/T | 0.369 | 0.349 | 0.751 | 0.216 |
|  |  | 187877807 | rs2228243 | A/G | 0.209 | 0.21 | 0.808 | 0.916 |
|  |  | 187878130 | rs1042445 | C/T | 0.237 | 0.226 | 0.502 | 0.452 |
|  | KNG1^(D4)^ | 187910034 | rs7624355 | C/T | 0.174 | 0.172 | 0.519 | 0.84 |
|  |  | 187910755 | rs10513803 | T/C | 0.39 | 0.412 | 0.51 | 0.186 |
|  |  | 187914126 | rs11918289 | G/A | 0.296 | 0.314 | 0.849 | 0.221 |
|  |  | 187916956 | rs3821815 | T/C | 0.294 | 0.331 | 0.024 | 0.014 |
|  |  | 187919092 | rs1851665 | A/G | 0.297 | 0.333 | 0.032 | 0.017 |
|  |  | 187921867 | rs1621816 | T/C | 0.302 | 0.303 | 0.391 | 0.934 |
|  |  | 187922692 | rs1403694 | T/C | 0.39 | 0.408 | 0.172 | 0.258 |
|  |  | 187925712 | rs1656922 | C/T | 0.452 | 0.495 | 0.027 | 9.71 10^-3^ |
|  |  | 187925944 | rs166479 | C/T | 0.451 | 0.496 | 0.021 | 7.59 10^-3^ |
|  |  | 187927746 | rs2304456 | T/G | 0.106 | 0.13 | 0.03 | 0.024 |
|  |  | 187928781 | rs5030102 | T/G | 0.131 | 0.119 | 0.69 | 0.265 |
|  |  | 187929741 | rs266723 | A/C | 0.449 | 0.471 | 0.162 | 0.195 |
|  |  | 187932610 | rs1648700 | T/C | 0.368 | 0.402 | 0.06 | 0.036 |
|  |  | 187933930 | rs4686799 | C/T | 0.201 | 0.227 | 0.191 | 0.05 |
|  |  | 187935611 | rs5030060 | C/T | 0.345 | 0.309 | 0.034 | 0.018 |
|  |  | 187936874 | rs5030062 | A/C | 0.412 | 0.371 | 9.96 10^-3^ | 0.01 |
|  |  | 187942621 | rs710446 | T/C | 0.454 | 0.41 | 1.17 10^-3^ | 6.43 10^-3^ |
|  |  | 187946037 | rs1972703 | G/A | 0.119 | 0.13 | 0.154 | 0.31 |
|  |  | 187946749 | rs6807774 | G/A | 0.495 | 0.477 | 0.127 | 0.103 |
|  |  | 187946801 | rs6796803 | C/T | 0.222 | 0.235 | 0.621 | 0.328 |
|  |  | 187948946 | rs1656966 | G/A | 0.139 | 0.151 | 0.633 | 0.289 |
|  |  | 187953880 | rs822373 | A/G | 0.403 | 0.38 | 0.167 | 0.15 |
| 4 | FGB ^(B)^ | 155700739 | rs4508864 | C/T | 0.196 | 0.208 | 0.189 | 0.379 |
|  |  | 155708271 | rs6056 | C/T | 0.178 | 0.187 | 0.257 | 0.484 |
|  |  | 155711209 | rs4220 | G/A | 0.178 | 0.187 | 0.253 | 0.458 |
|  |  | 155714983 | rs4463047 | T/C | 0.151 | 0.123 | 0.02 | 0.015 |
|  | FGA  ^(B)^ | 155720638 | rs6825454 | T/C | 0.299 | 0.228 | 1.32 10^-9^ | 1.89 10^-6^ |
|  |  | 155721563 | rs4308349 | A/G | 0.115 | 0.13 | 0.108 | 0.187 |
|  |  | 155724398 | rs2070022 | G/A | 0.145 | 0.171 | 8.26 10^-3^ | 0.029 |
|  |  | 155729764 | rs2070016 | A/G | 0.142 | 0.157 | 0.074 | 0.219 |
|  |  | 155733316 | rs2070006 | C/T | 0.415 | 0.355 | 6.05 10^-6^ | 2.13 10^-4^ |
|  | FGG ^(B)^ | 155744726 | rs2066865 | G/A | 0.28 | 0.209 | 1.17 10^-10^ | 6.98 10^-7^ |
|  |  | 155748924 | rs2066860 | C/T | 0.033 | 0.033 | 0.79 | 0.968 |
|  |  | 155753858 | rs1800792 | T/C | 0.447 | 0.477 | 0.056 | 0.069 |
|  |  | 155760739 | rs12648395 | T/C | 0.262 | 0.307 | 2.83 10^-4^ | 2.38 10^-3^ |
|  |  | 155767903 | rs13137121 | A/G | 0.377 | 0.374 | 0.563 | 0.86 |
|  |  | 155768804 | rs10050268 | C/T | 0.128 | 0.127 | 0.789 | 0.914 |
|  |  | 155795734 | rs1074801 | C/A | 0.483 | 0.493 | 0.958 | 0.536 |
|  |  | 155799868 | rs6828439 | C/T | 0.052 | 0.053 | 0.775 | 0.963 |
|  |  | 155809510 | rs10011108 | A/C | 0.053 | 0.053 | 0.647 | 0.922 |
|  |  | 155815971 | rs767513 | A/G | 0.482 | 0.491 | 1 | 0.55 |
|  | CYP4V2 ^(C)^ | 187353221 | rs7684025 | G/A | 0.451 | 0.406 | 2.39 10^-4^ | 5.94 10^-3^ |
|  |  | 187357205 | rs13146272 | A/C | 0.351 | 0.39 | 2.17 10^-4^ | 0.015 |
|  |  | 187359313 | rs3736455 | G/T | 0.326 | 0.368 | 1.84 10^-5^ | 7.42 10^-3^ |
|  |  | 187359349 | rs3736456 | T/C | 0.064 | 0.059 | 0.138 | 0.572 |
|  |  | 187364811 | rs1473597 | A/G | 0.418 | 0.432 | 0.348 | 0.373 |
|  |  | 187366989 | rs2276917 | A/G | 0.415 | 0.432 | 0.329 | 0.311 |
|  |  | 187368378 | rs9995366 | C/T | 0.079 | 0.114 | 8.90 10^-5^ | 2.81 10^-4^ |
|  |  | 187368498 | rs2102575 | A/G | 0.07 | 0.108 | 1.06 10^-5^ | 4.81 10^-4^ |
|  | KLKB1 ^(C)^ | 187385065 | rs4253236 | C/T | 0.355 | 0.394 | 5.03 10^-3^ | 0.015 |
|  |  | 187385381 | rs4253238 | T/C | 0.473 | 0.485 | 5.64 10-3 | 0.012 |
|  |  | 187385469 | rs4253239 | C/T | 0.185 | 0.197 | 0.163 | 0.367 |
|  |  | 187386534 | rs1912826 | A/G | 0.476 | 0.485 | 8.81 10^-3^ | 0.019 |
|  |  | 187387800 | rs1511802 | T/C | 0.399 | 0.363 | 7.88 10^-3^ | 0.028 |
|  |  | 187410006 | rs4253301 | T/G | 0.117 | 0.119 | 0.962 | 0.829 |
|  |  | 187413828 | rs4241821 | C/T | 0.17 | 0.197 | 0.043 | 0.038 |
|  |  | 187414808 | rs3775302 | A/G | 0.128 | 0.125 | 0.982 | 0.797 |
|  |  | 187415467 | rs4253325 | G/A | 0.094 | 0.101 | 0.13 | 0.483 |
|  |  | 187416129 | rs4253331 | T/C | 0.099 | 0.084 | 0.078 | 0.129 |
|  |  | 187416204 | rs925453 | C/T | 0.286 | 0.314 | 0.074 | 0.065 |
|  |  | 187419654 | rs11132383 | T/C | 0.461 | 0.479 | 0.025 | 0.294 |
|  | F11 ^(C)^ | 187423999 | rs3822056 | G/T | 0.082 | 0.093 | 0.277 | 0.248 |
|  |  | 187424563 | rs925451 | G/A | 0.456 | 0.398 | 1.27 10^-6^ | 4.18 10^-4^ |
|  |  | 187436491 | rs4253418 | G/A | 0.023 | 0.05 | 2.57 10^-6^ | 9.97 10^-6^ |
|  |  | 187436882 | rs4253419 | A/G | 0.182 | 0.199 | 0.128 | 0.209 |
|  |  | 187437995 | rs2241817 | A/G | 0.376 | 0.383 | 0.193 | 0.642 |
|  |  | 187443174 | rs3822058 | G/A | 0.377 | 0.384 | 0.187 | 0.676 |
|  |  | 187446553 | rs5966 | A/G | 0.049 | 0.052 | 0.285 | 0.641 |
|  |  | 187446696 | rs5971 | G/T | 0.049 | 0.052 | 0.318 | 0.687 |
|  |  | 187449128 | rs11938564 | T/G | 0.229 | 0.25 | 0.049 | 0.155 |
|  |  | 187452010 | rs13145616 | C/A | 0.146 | 0.133 | 0.634 | 0.263 |
|  |  | 187454632 | rs12500151 | A/G | 0.292 | 0.309 | 0.131 | 0.288 |
|  |  | 187455812 | rs6848311 | G/T | 0.171 | 0.215 | 1.83 10^-4^ | 6.25 10^-4^ |
|  |  | 187459594 | rs10029715 | T/C | 0.115 | 0.172 | 3.20 10^-9^ | 3.08 10^-7^ |
|  |  | 187463667 | rs1008728 | T/C | 0.344 | 0.403 | 5.76 10^-7^ | 2.81 10^-4^ |
|  |  | 187467731 | rs13133050 | C/A | 0.293 | 0.344 | 1.49 10^-5^ | 1.03 10^-3^ |
|  |  | 187470832 | rs12331264 | T/C | 0.331 | 0.345 | 0.263 | 0.388 |
|  |  | 187472992 | rs10020303 | A/G | 0.243 | 0.216 | 0.084 | 0.056 |
|  |  | 187476741 | rs7687352 | A/G | 0.462 | 0.494 | 0.238 | 0.06 |
|  |  | 187478491 | rs7700014 | T/C | 0.373 | 0.354 | 0.203 | 0.236 |
|  |  | 187484083 | rs7349633 | G/T | 0.243 | 0.242 | 0.954 | 0.954 |
|  |  | 187488854 | rs11132389 | A/G | 0.372 | 0.399 | 0.042 | 0.098 |
|  |  | 187493998 | rs6829128 | C/T | 0.346 | 0.366 | 0.068 | 0.199 |
|  |  | 187499483 | rs10032695 | T/G | 0.346 | 0.32 | 0.035 | 0.099 |
|  |  | 187508041 | rs7672650 | T/C | 0.352 | 0.348 | 0.923 | 0.812 |
|  |  | 187513906 | rs10015908 | C/T | 0.205 | 0.219 | 0.51 | 0.307 |
|  |  | 187514158 | rs10016252 | A/G | 0.286 | 0.274 | 0.302 | 0.428 |
| 5 | F12 ^(A)^ | 176775080 | rs2731672 | C/T | 0.211 | 0.229 | 0.471 | 0.197 |
| 6 | F13A1 ^(B)^ | 6021432 | rs6597185 | C/T | 0.489 | 0.5 | 0.711 | 0.498 |
|  |  | 6025435 | rs6910246 | A/G | 0.363 | 0.355 | 0.765 | 0.626 |
|  |  | 6026070 | rs11757860 | A/G | 0.244 | 0.264 | 0.22 | 0.17 |
|  |  | 6032973 | rs4960164 | A/C | 0.184 | 0.184 | 0.919 | 1 |
|  |  | 6033054 | rs17141697 | T/G | 0.144 | 0.137 | 0.462 | 0.49 |
|  |  | 6033331 | rs9504663 | C/A | 0.418 | 0.436 | 0.622 | 0.259 |
|  |  | 6038448 | rs6915313 | C/T | 0.292 | 0.293 | 0.757 | 0.924 |
|  |  | 6042535 | rs7755737 | A/G | 0.116 | 0.107 | 0.484 | 0.413 |
|  |  | 6059381 | rs2876006 | T/G | 0.244 | 0.265 | 0.171 | 0.14 |
|  |  | 6071844 | rs13201744 | C/A | 0.108 | 0.107 | 0.593 | 0.913 |
|  |  | 6074216 | rs2220053 | T/G | 0.278 | 0.297 | 0.392 | 0.207 |
|  |  | 6074504 | rs1482871 | A/G | 0.239 | 0.241 | 0.454 | 0.892 |
|  |  | 6076323 | rs1351656 | T/C | 0.268 | 0.261 | 0.805 | 0.658 |
|  |  | 6077478 | rs9502417 | T/C | 0.389 | 0.374 | 0.765 | 0.342 |
|  |  | 6079185 | rs13212444 | C/T | 0.071 | 0.065 | 0.498 | 0.476 |
|  |  | 6081282 | rs10484321 | T/G | 0.314 | 0.323 | 0.671 | 0.55 |
|  |  | 6082107 | rs2170618 | C/T | 0.251 | 0.247 | 0.778 | 0.786 |
|  |  | 6084568 | rs7772158 | C/A | 0.461 | 0.471 | 0.518 | 0.541 |
|  |  | 6090408 | rs3024486 | C/T | 0.058 | 0.071 | 0.25 | 0.106 |
|  |  | 6090691 | rs1050783 | C/T | 0.181 | 0.178 | 0.792 | 0.821 |
|  |  | 6095121 | rs3778361 | C/T | 0.071 | 0.068 | 0.717 | 0.69 |
|  |  | 6095737 | rs3799563 | C/T | 0.384 | 0.37 | 0.82 | 0.394 |
|  |  | 6096291 | rs4282440 | C/A | 0.251 | 0.246 | 0.722 | 0.715 |
|  |  | 6096818 | rs3024459 | T/C | 0.121 | 0.112 | 0.569 | 0.391 |
|  |  | 6097554 | rs13213508 | G/A | 0.072 | 0.069 | 0.758 | 0.711 |
|  |  | 6098001 | rs13206518 | T/C | 0.075 | 0.072 | 0.654 | 0.681 |
|  |  | 6098988 | rs9392751 | C/A | 0.131 | 0.126 | 0.758 | 0.604 |
|  |  | 6101326 | rs13200015 | G/A | 0.076 | 0.075 | 0.91 | 0.947 |
|  |  | 6102139 | rs13193648 | T/C | 0.254 | 0.245 | 0.446 | 0.51 |
|  |  | 6102956 | rs2326708 | G/A | 0.219 | 0.234 | 0.149 | 0.282 |
|  |  | 6103148 | rs381061 | C/T | 0.276 | 0.272 | 0.766 | 0.809 |
|  |  | 6104048 | rs4960171 | C/T | 0.451 | 0.454 | 0.568 | 0.855 |
|  |  | 6106108 | rs6927469 | T/G | 0.235 | 0.241 | 0.859 | 0.701 |
|  |  | 6108087 | rs6911594 | A/G | 0.482 | 0.491 | 0.42 | 0.596 |
|  |  | 6108491 | rs13202850 | A/G | 0.151 | 0.144 | 0.673 | 0.501 |
|  |  | 6110467 | rs434602 | T/C | 0.371 | 0.384 | 0.607 | 0.439 |
|  |  | 6110781 | rs440330 | T/C | 0.045 | 0.048 | 0.982 | 0.735 |
|  |  | 6110833 | rs922866 | T/C | 0.165 | 0.172 | 0.406 | 0.613 |
|  |  | 6113193 | rs900401 | C/T | 0.211 | 0.216 | 0.707 | 0.728 |
|  |  | 6114217 | rs3823193 | A/G | 0.208 | 0.192 | 0.384 | 0.222 |
|  |  | 6114655 | rs7769202 | T/C | 0.365 | 0.349 | 0.204 | 0.335 |
|  |  | 6115448 | rs1609584 | T/C | 0.222 | 0.244 | 0.15 | 0.122 |
|  |  | 6116671 | rs755656 | T/C | 0.495 | 0.468 | 0.166 | 0.1 |
|  |  | 6117301 | rs406238 | C/A | 0.338 | 0.369 | 0.341 | 0.047 |
|  |  | 6118986 | rs786735 | T/C | 0.207 | 0.188 | 0.749 | 0.145 |
|  |  | 6119865 | rs5982 | G/A | 0.214 | 0.21 | 0.586 | 0.763 |
|  |  | 6120173 | rs3024444 | C/T | 0.104 | 0.11 | 0.39 | 0.528 |
|  |  | 6120590 | rs407447 | T/C | 0.335 | 0.302 | 0.282 | 0.032 |
|  |  | 6120974 | rs11969912 | G/A | 0.327 | 0.34 | 0.908 | 0.404 |
|  |  | 6121143 | rs12210959 | T/C | 0.247 | 0.249 | 0.61 | 0.878 |
|  |  | 6121624 | rs7774391 | G/A | 0.32 | 0.307 | 0.358 | 0.385 |
|  |  | 6122043 | rs3778354 | T/G | 0.463 | 0.468 | 0.879 | 0.765 |
|  |  | 6123327 | rs3116567 | C/T | 0.16 | 0.166 | 0.69 | 0.639 |
|  |  | 6124881 | rs3778353 | T/C | 0.303 | 0.322 | 0.495 | 0.209 |
|  |  | 6126525 | rs3024443 | C/T | 0.122 | 0.116 | 0.802 | 0.553 |
|  |  | 6127604 | rs2274393 | C/T | 0.237 | 0.23 | 0.594 | 0.623 |
|  |  | 6129351 | rs373695 | A/G | 0.074 | 0.089 | 0.339 | 0.086 |
|  |  | 6129818 | rs3799558 | C/A | 0.417 | 0.432 | 0.519 | 0.362 |
|  |  | 6139966 | rs3823191 | C/T | 0.486 | 0.495 | 0.259 | 0.253 |
|  |  | 6140059 | rs11964160 | A/G | 0.142 | 0.137 | 0.992 | 0.701 |
|  |  | 6144881 | rs4959374 | G/A | 0.411 | 0.424 | 0.7 | 0.429 |
|  |  | 6145874 | rs6924490 | C/T | 0.174 | 0.162 | 0.928 | 0.35 |
|  |  | 6147733 | rs1482868 | C/T | 0.356 | 0.339 | 0.443 | 0.29 |
|  |  | 6149340 | rs9392757 | G/A | 0.036 | 0.038 | 0.704 | 0.675 |
|  |  | 6150841 | rs2085575 | C/A | 0.407 | 0.419 | 0.736 | 0.485 |
|  |  | 6156418 | rs3024422 | A/G | 0.17 | 0.165 | 0.572 | 0.669 |
|  |  | 6165028 | rs1993552 | A/G | 0.209 | 0.201 | 0.446 | 0.522 |
|  |  | 6167736 | rs3024415 | T/C | 0.391 | 0.407 | 0.564 | 0.312 |
|  |  | 6168293 | rs3024409 | C/A | 0.312 | 0.303 | 0.503 | 0.539 |
|  |  | 6173405 | rs1742930 | G/A | 0.125 | 0.123 | 0.424 | 0.813 |
|  |  | 6183594 | rs13217372 | T/C | 0.187 | 0.206 | 0.061 | 0.135 |
|  |  | 6187592 | rs17376901 | G/T | 0.196 | 0.212 | 0.088 | 0.222 |
|  |  | 6189123 | rs1613671 | G/A | 0.448 | 0.457 | 0.265 | 0.6 |
|  |  | 6191983 | rs1267843 | T/C | 0.46 | 0.447 | 0.976 | 0.431 |
|  |  | 6194275 | rs3024391 | C/T | 0.364 | 0.355 | 0.694 | 0.59 |
|  |  | 6194619 | rs3024389 | G/A | 0.397 | 0.404 | 0.934 | 0.676 |
|  |  | 6194644 | rs3024388 | C/T | 0.234 | 0.237 | 0.522 | 0.857 |
|  |  | 6194701 | rs3024387 | T/C | 0.399 | 0.405 | 0.941 | 0.709 |
|  |  | 6195500 | rs3024377 | T/C | 0.143 | 0.133 | 0.132 | 0.367 |
|  |  | 6195966 | rs3024370 | G/A | 0.287 | 0.29 | 0.497 | 0.827 |
|  |  | 6200850 | rs9379022 | A/G | 0.244 | 0.251 | 0.635 | 0.638 |
|  |  | 6201832 | rs7766109 | A/G | 0.492 | 0.472 | 0.321 | 0.237 |
|  |  | 6203629 | rs9504738 | A/G | 0.065 | 0.071 | 0.992 | 0.46 |
|  |  | 6205748 | rs3901123 | C/T | 0.066 | 0.063 | 0.989 | 0.676 |
|  |  | 6210193 | rs10484322 | C/A | 0.111 | 0.127 | 0.487 | 0.131 |
|  |  | 6212303 | rs3024358 | G/T | 0.195 | 0.204 | 0.33 | 0.491 |
|  |  | 6216061 | rs17374745 | G/A | 0.191 | 0.202 | 0.304 | 0.398 |
|  |  | 6217185 | rs10484323 | T/C | 0.066 | 0.072 | 0.929 | 0.523 |
|  |  | 6219083 | rs3863222 | A/G | 0.28 | 0.291 | 0.128 | 0.469 |
|  |  | 6219569 | rs3851514 | C/T | 0.322 | 0.306 | 0.225 | 0.305 |
|  |  | 6219638 | rs12529193 | G/T | 0.187 | 0.202 | 0.12 | 0.264 |
|  |  | 6219946 | rs9328347 | G/A | 0.109 | 0.13 | 0.388 | 0.05 |
|  |  | 6220931 | rs9328348 | T/C | 0.309 | 0.321 | 0.281 | 0.435 |
|  |  | 6225287 | rs4959377 | T/C | 0.373 | 0.349 | 0.076 | 0.132 |
|  |  | 6227777 | rs9405911 | A/G | 0.488 | 0.495 | 0.652 | 0.313 |
|  |  | 6227942 | rs12664620 | A/G | 0.23 | 0.236 | 0.634 | 0.667 |
|  |  | 6228665 | rs749005 | G/T | 0.164 | 0.167 | 0.223 | 0.832 |
|  |  | 6229124 | rs9502429 | C/T | 0.499 | 0.484 | 0.452 | 0.316 |
|  |  | 6231853 | rs1993553 | T/C | 0.205 | 0.202 | 0.42 | 0.819 |
|  |  | 6232615 | rs6906354 | G/A | 0.228 | 0.218 | 0.716 | 0.482 |
|  |  | 6235002 | rs11243069 | G/A | 0.127 | 0.129 | 0.613 | 0.83 |
|  |  | 6239058 | rs9504743 | A/G | 0.241 | 0.23 | 0.83 | 0.417 |
|  |  | 6249530 | rs1742932 | A/G | 0.249 | 0.283 | 0.229 | 0.026 |
|  |  | 6250476 | rs2295753 | C/A | 0.153 | 0.142 | 0.931 | 0.325 |
|  |  | 6251404 | rs714408 | A/G | 0.365 | 0.36 | 0.881 | 0.768 |
|  |  | 6256700 | rs2755416 | G/A | 0.338 | 0.325 | 0.458 | 0.418 |
|  |  | 6259642 | rs3024342 | T/C | 0.181 | 0.204 | 0.344 | 0.076 |
|  |  | 6261801 | rs11243078 | T/G | 0.113 | 0.107 | 0.941 | 0.539 |
|  |  | 6263794 | rs5985 | C/A | 0.226 | 0.253 | 0.333 | 0.055 |
|  |  | 6264534 | rs3024471 | A/G | 0.028 | 0.025 | 0.578 | 0.663 |
|  |  | 6265807 | rs2815822 | G/T | 0.121 | 0.133 | 0.184 | 0.288 |
|  |  | 6267874 | rs3024304 | G/T | 0.281 | 0.248 | 0.398 | 0.024 |
|  |  | 6267977 | rs1267858 | C/T | 0.084 | 0.064 | 0.115 | 0.026 |
|  |  | 6268419 | rs17379383 | C/T | 0.02 | 0.028 | 0.033 | 0.101 |
|  |  | 6271857 | rs1781789 | T/C | 0.13 | 0.115 | 0.676 | 0.188 |
|  |  | 6273145 | rs11755846 | A/G | 0.053 | 0.074 | 0.062 | 8.15 10-3 |
|  |  | 6276443 | rs1742917 | T/G | 0.159 | 0.167 | 0.832 | 0.521 |
|  |  | 6276935 | rs1318606 | T/C | 0.402 | 0.425 | 0.559 | 0.162 |
|  |  | 6281921 | rs3863223 | G/T | 0.379 | 0.387 | 0.778 | 0.616 |
|  |  | 6282505 | rs11243081 | C/T | 0.307 | 0.32 | 0.644 | 0.402 |
|  |  | 6284376 | rs1674067 | G/A | 0.128 | 0.116 | 0.627 | 0.282 |
|  |  | 6284445 | rs10498668 | T/C | 0.072 | 0.068 | 0.919 | 0.619 |
|  | HIVEP1 ^(C)^ | 12046658 | rs6904014 | C/T | 0.031 | 0.026 | 0.349 | 0.302 |
|  |  | 12046783 | rs169715 | A/G | 0.06 | 0.036 | 4.78 10^-4^ | 8.03 10^-4^ |
|  |  | 12056122 | rs1205887 | C/T | 0.176 | 0.155 | 0.787 | 0.083 |
|  |  | 12060831 | rs10947670 | A/G | 0.31 | 0.265 | 0.122 | 2.74 10^-3^ |
|  |  | 12067874 | rs6941983 | T/C | 0.24 | 0.197 | 0.042 | 2.08 10^-3^ |
|  |  | 12068692 | rs6927024 | C/T | 0.24 | 0.198 | 0.044 | 2.25 10^-3^ |
|  |  | 12069956 | rs6921943 | A/C | 0.313 | 0.269 | 0.148 | 4.40 10^-3^ |
|  |  | 12070732 | rs12193434 | T/C | 0.076 | 0.074 | 0.447 | 0.856 |
|  |  | 12071208 | rs186017 | A/G | 0.146 | 0.111 | 4.61 10^-3^ | 1.56 10^-3^ |
|  |  | 12073684 | rs7775818 | C/T | 0.067 | 0.064 | 0.848 | 0.634 |
|  |  | 12077217 | rs11963539 | C/T | 0.136 | 0.105 | 0.02 | 0.00397 |
|  |  | 12080695 | rs9296224 | G/A | 0.102 | 0.092 | 0.911 | 0.293 |
|  |  | 12080728 | rs913021 | T/C | 0.376 | 0.375 | 0.601 | 0.971 |
|  |  | 12082826 | rs1409288 | G/A | 0.271 | 0.281 | 0.574 | 0.524 |
|  |  | 12084725 | rs9470631 | A/G | 0.132 | 0.1 | 0.013 | 3.23 10^-3^ |
|  |  | 12090447 | rs4711507 | T/C | 0.207 | 0.224 | 0.408 | 0.224 |
|  |  | 12093319 | rs11752244 | G/A | 0.131 | 0.101 | 0.015 | 3.90 10^-3^ |
|  |  | 12094523 | rs11961287 | T/G | 0.133 | 0.101 | 0.015 | 2.97 10^-3^ |
|  |  | 12128227 | rs6925772 | C/T | 0.197 | 0.204 | 0.862 | 0.612 |
|  |  | 12135388 | rs17697699 | A/G | 0.304 | 0.349 | 3.22 10^-3^ | 3.81 10^-3^ |
|  |  | 12137410 | rs2327506 | C/T | 0.477 | 0.431 | 0.074 | 5.69 10^-3^ |
|  |  | 12141237 | rs9296237 | T/C | 0.063 | 0.048 | 0.358 | 0.047 |
|  |  | 12143315 | rs12525800 | C/A | 0.474 | 0.5 | 0.433 | 0.121 |
|  |  | 12144643 | rs17676598 | G/T | 0.256 | 0.279 | 0.065 | 0.116 |
|  |  | 12150191 | rs12234132 | A/C | 0.145 | 0.121 | 0.041 | 0.032 |
|  |  | 12152040 | rs9470843 | T/C | 0.276 | 0.247 | 0.071 | 0.047 |
|  |  | 12156113 | rs9349068 | G/A | 0.124 | 0.111 | 0.231 | 0.202 |
|  |  | 12156813 | rs1570989 | G/A | 0.227 | 0.237 | 0.563 | 0.449 |
|  |  | 12171854 | rs6930779 | G/A | 0.068 | 0.045 | 1.81 10^-3^ | 2.23 10^-3^ |
|  |  | 12172004 | rs7749026 | A/G | 0.401 | 0.43 | 1.83 10^-3^ | 0.068 |
|  |  | 12175795 | rs17608729 | G/A | 0.179 | 0.183 | 0.185 | 0.759 |
|  |  | 12177683 | rs4714170 | A/G | 0.283 | 0.264 | 0.156 | 0.195 |
|  |  | 12179270 | rs6916070 | T/C | 0.273 | 0.271 | 0.186 | 0.9 |
|  |  | 12179374 | rs16872237 | C/T | 0.139 | 0.143 | 0.313 | 0.71 |
|  |  | 12185708 | rs7770934 | G/A | 0.159 | 0.123 | 0.014 | 2.47 10^-3^ |
|  |  | 12186698 | rs2327508 | G/A | 0.392 | 0.404 | 0.37 | 0.482 |
|  |  | 12186758 | rs2327509 | A/G | 0.225 | 0.252 | 0.035 | 0.053 |
|  |  | 12188531 | rs12176308 | A/G | 0.056 | 0.046 | 0.022 | 0.14 |
|  |  | 12188635 | rs9394517 | G/T | 0.056 | 0.045 | 0.019 | 0.138 |
|  |  | 12190200 | rs9380764 | C/T | 0.056 | 0.046 | 0.023 | 0.154 |
|  |  | 12194812 | rs4467770 | A/G | 0.291 | 0.308 | 0.123 | 0.273 |
|  |  | 12195878 | rs10947756 | G/A | 0.356 | 0.38 | 0.021 | 0.125 |
|  |  | 12195974 | rs17608937 | G/T | 0.202 | 0.201 | 0.103 | 0.992 |
|  |  | 12197891 | rs6939913 | T/C | 0.394 | 0.427 | 6.98 10^-3^ | 0.043 |
|  |  | 12207264 | rs9380781 | A/G | 0.373 | 0.404 | 0.016 | 0.056 |
|  |  | 12210601 | rs7768491 | T/C | 0.116 | 0.119 | 0.204 | 0.822 |
|  |  | 12219855 | rs12213481 | G/A | 0.061 | 0.072 | 0.528 | 0.161 |
|  |  | 12225541 | rs2327514 | G/A | 0.133 | 0.143 | 0.098 | 0.398 |
|  |  | 12230160 | rs2228210 | A/G | 0.372 | 0.402 | 0.022 | 0.068 |
|  |  | 12231235 | rs2228220 | A/G | 0.122 | 0.085 | 3.70 10^-4^ | 2.57 10^-4^ |
|  |  | 12232841 | rs2228213 | G/A | 0.369 | 0.396 | 0.033 | 0.095 |
|  |  | 12233758 | rs1126472 | A/G | 0.142 | 0.103 | 1.91 10^-3^ | 3.16 10^-4^ |
|  |  | 12243420 | rs3777772 | A/G | 0.147 | 0.158 | 0.608 | 0.352 |
|  |  | 12260833 | rs3777758 | G/A | 0.065 | 0.049 | 0.041 | 0.03 |
|  |  | 12267098 | rs7747677 | G/A | 0.234 | 0.238 | 0.836 | 0.796 |
|  |  | 12273349 | rs219950 | C/T | 0.332 | 0.328 | 0.354 | 0.771 |
|  |  | 12274485 | rs16872348 | T/C | 0.184 | 0.191 | 0.778 | 0.583 |
|  |  | 12279143 | rs9394605 | G/A | 0.334 | 0.372 | 5.31 10^-3^ | 0.015 |
|  |  | 12281743 | rs10947819 | T/G | 0.098 | 0.11 | 0.067 | 0.245 |
|  |  | 12291036 | rs10947826 | G/A | 0.319 | 0.351 | 0.021 | 0.042 |
|  |  | 12291938 | rs11755555 | G/A | 0.098 | 0.108 | 0.052 | 0.368 |
|  |  | 12300174 | rs2876257 | T/C | 0.4 | 0.415 | 0.777 | 0.344 |
|  |  | 12304896 | rs1040992 | G/T | 0.205 | 0.206 | 0.904 | 0.963 |
|  |  | 12309750 | rs9380902 | A/G | 0.267 | 0.276 | 0.326 | 0.553 |
|  |  | 12311249 | rs6458125 | A/G | 0.472 | 0.483 | 0.356 | 0.476 |
|  |  | 12320038 | rs220000 | A/G | 0.096 | 0.091 | 0.643 | 0.602 |
|  |  | 12320356 | rs405742 | G/A | 0.488 | 0.49 | 0.344 | 0.9 |
|  |  | 12321683 | rs220009 | T/C | 0.444 | 0.467 | 0.503 | 0.182 |
|  |  | 12322312 | rs9367034 | G/A | 0.27 | 0.277 | 0.826 | 0.626 |
|  |  | 12324462 | rs12662776 | T/G | 0.296 | 0.312 | 0.705 | 0.291 |
|  |  | 12324632 | rs1794856 | G/A | 0.349 | 0.369 | 0.695 | 0.212 |
|  |  | 12325106 | rs12208557 | G/T | 0.084 | 0.091 | 0.741 | 0.475 |
|  |  | 12325408 | rs7356945 | C/T | 0.322 | 0.306 | 0.929 | 0.289 |
|  |  | 12325847 | rs4714277 | A/G | 0.343 | 0.35 | 1 | 0.689 |
|  |  | 12325985 | rs6908010 | G/T | 0.425 | 0.438 | 0.67 | 0.413 |
|  |  | 12326828 | rs9471234 | G/A | 0.343 | 0.349 | 0.984 | 0.741 |
|  |  | 12327771 | rs12662518 | C/T | 0.233 | 0.213 | 0.556 | 0.149 |
|  |  | 12332517 | rs2327538 | C/T | 0.459 | 0.469 | 0.674 | 0.557 |
|  |  | 12332702 | rs10947850 | T/C | 0.074 | 0.073 | 0.752 | 0.901 |
|  |  | 12335502 | rs6458136 | T/C | 0.451 | 0.452 | 0.742 | 0.944 |
|  | BAI3 ^(C)^ | 69407020 | rs2802699 | C/A | 0.365 | 0.395 | 0.036 | 0.059 |
|  |  | 69414389 | rs9360356 | G/T | 0.153 | 0.143 | 0.408 | 0.417 |
|  |  | 69435338 | rs2585622 | G/A | 0.457 | 0.429 | 0.195 | 0.089 |
|  |  | 69435704 | rs2254654 | A/G | 0.49 | 0.467 | 0.269 | 0.165 |
|  |  | 69473052 | rs1983623 | T/C | 0.171 | 0.184 | 0.945 | 0.295 |
|  |  | 69478185 | rs2206835 | G/A | 0.151 | 0.147 | 0.82 | 0.76 |
|  |  | 69479439 | rs2802679 | A/G | 0.304 | 0.288 | 0.845 | 0.313 |
|  |  | 69486083 | rs12206222 | A/G | 0.438 | 0.414 | 0.333 | 0.136 |
|  |  | 69495828 | rs9363963 | G/A | 0.169 | 0.184 | 0.884 | 0.228 |
|  |  | 69496207 | rs9294806 | T/C | 0.19 | 0.2 | 0.649 | 0.442 |
|  |  | 69516351 | rs10945140 | T/C | 0.187 | 0.198 | 0.71 | 0.411 |
|  |  | 69516663 | rs9346237 | A/G | 0.324 | 0.329 | 0.379 | 0.738 |
|  |  | 69517695 | rs6908159 | G/A | 0.334 | 0.34 | 0.275 | 0.687 |
|  |  | 69521107 | rs10484791 | T/C | 0.481 | 0.464 | 0.466 | 0.303 |
|  |  | 69523490 | rs12211047 | T/G | 0.223 | 0.23 | 0.778 | 0.584 |
|  |  | 69524871 | rs2073135 | A/G | 0.279 | 0.267 | 0.563 | 0.431 |
|  |  | 69531215 | rs9346239 | A/G | 0.389 | 0.398 | 0.808 | 0.602 |
|  |  | 69537129 | rs1523932 | C/T | 0.331 | 0.336 | 0.396 | 0.75 |
|  |  | 69537349 | rs1357618 | G/T | 0.183 | 0.2 | 0.954 | 0.197 |
|  |  | 69540429 | rs1523947 | A/G | 0.234 | 0.25 | 0.822 | 0.247 |
|  |  | 69543925 | rs2204285 | G/A | 0.184 | 0.199 | 0.969 | 0.242 |
|  |  | 69574954 | rs2177448 | G/A | 0.232 | 0.248 | 0.876 | 0.27 |
|  |  | 69576670 | rs9454623 | T/G | 0.157 | 0.15 | 0.953 | 0.555 |
|  |  | 69578699 | rs582962 | A/G | 0.306 | 0.311 | 0.979 | 0.713 |
|  |  | 69582086 | rs9360362 | G/A | 0.21 | 0.226 | 0.717 | 0.233 |
|  |  | 69588678 | rs2022212 | A/G | 0.123 | 0.131 | 0.421 | 0.452 |
|  |  | 69597835 | rs514518 | A/C | 0.405 | 0.42 | 0.158 | 0.364 |
|  |  | 69602624 | rs1008234 | G/A | 0.399 | 0.372 | 0.29 | 0.09 |
|  |  | 69604733 | rs544495 | G/A | 0.092 | 0.096 | 0.649 | 0.679 |
|  |  | 69604927 | rs1403923 | G/A | 0.212 | 0.192 | 0.18 | 0.141 |
|  |  | 69605819 | rs483076 | G/A | 0.092 | 0.096 | 0.587 | 0.72 |
|  |  | 69606618 | rs493768 | C/T | 0.194 | 0.203 | 0.241 | 0.506 |
|  |  | 69616807 | rs13207774 | A/G | 0.191 | 0.185 | 0.849 | 0.613 |
|  |  | 69617706 | rs500125 | A/G | 0.499 | 0.472 | 0.164 | 0.097 |
|  |  | 69621367 | rs507326 | G/T | 0.499 | 0.473 | 0.193 | 0.113 |
|  |  | 69627774 | rs6455306 | C/T | 0.084 | 0.086 | 0.502 | 0.822 |
|  |  | 69638712 | rs551216 | A/G | 0.302 | 0.321 | 0.37 | 0.19 |
|  |  | 69648601 | rs562537 | T/C | 0.302 | 0.32 | 0.406 | 0.21 |
|  |  | 69649257 | rs478789 | G/A | 0.196 | 0.203 | 0.29 | 0.583 |
|  |  | 69654724 | rs12209015 | G/A | 0.2 | 0.206 | 0.559 | 0.66 |
|  |  | 69662403 | rs1523936 | T/C | 0.195 | 0.205 | 0.21 | 0.471 |
|  |  | 69663469 | rs1916767 | A/G | 0.405 | 0.375 | 0.232 | 0.057 |
|  |  | 69667002 | rs2342761 | G/A | 0.404 | 0.428 | 0.748 | 0.143 |
|  |  | 69671649 | rs10485437 | G/A | 0.405 | 0.373 | 0.205 | 0.048 |
|  |  | 69671889 | rs10485436 | A/G | 0.135 | 0.132 | 0.922 | 0.774 |
|  |  | 69672336 | rs10485435 | G/T | 0.292 | 0.302 | 0.789 | 0.47 |
|  |  | 69675201 | rs9294813 | A/C | 0.405 | 0.373 | 0.217 | 0.049 |
|  |  | 69675728 | rs1283951 | C/T | 0.074 | 0.08 | 0.9 | 0.49 |
|  |  | 69695225 | rs12527597 | C/T | 0.111 | 0.125 | 0.911 | 0.171 |
|  |  | 69703965 | rs2342763 | G/A | 0.131 | 0.135 | 0.49 | 0.736 |
|  |  | 69708145 | rs1932613 | C/T | 0.208 | 0.214 | 0.654 | 0.699 |
|  |  | 69709715 | rs7748808 | A/G | 0.113 | 0.126 | 0.921 | 0.228 |
|  |  | 69711371 | rs7759377 | T/C | 0.112 | 0.127 | 0.849 | 0.169 |
|  |  | 69714971 | rs13193787 | C/T | 0.24 | 0.261 | 0.417 | 0.152 |
|  |  | 69717562 | rs1932616 | C/T | 0.116 | 0.117 | 0.347 | 0.913 |
|  |  | 69720601 | rs1889879 | A/C | 0.361 | 0.364 | 0.881 | 0.811 |
|  |  | 69723405 | rs1932618 | G/A | 0.139 | 0.131 | 0.342 | 0.44 |
|  |  | 69724193 | rs3823061 | G/A | 0.124 | 0.11 | 0.235 | 0.211 |
|  |  | 69728760 | rs3798971 | A/G | 0.33 | 0.326 | 0.705 | 0.777 |
|  |  | 69731591 | rs9446078 | A/G | 0.32 | 0.303 | 0.803 | 0.272 |
|  |  | 69731968 | rs2880587 | G/A | 0.348 | 0.347 | 0.703 | 0.957 |
|  |  | 69732588 | rs12191480 | T/C | 0.221 | 0.23 | 0.367 | 0.473 |
|  |  | 69734638 | rs1953613 | G/A | 0.351 | 0.368 | 0.766 | 0.278 |
|  |  | 69734838 | rs3798977 | A/G | 0.338 | 0.329 | 0.982 | 0.557 |
|  |  | 69735685 | rs9454661 | C/A | 0.296 | 0.315 | 0.977 | 0.21 |
|  |  | 69738381 | rs3798979 | T/C | 0.45 | 0.425 | 0.661 | 0.132 |
|  |  | 69740195 | rs9454664 | G/T | 0.312 | 0.3 | 0.923 | 0.414 |
|  |  | 69741571 | rs2275209 | T/G | 0.135 | 0.152 | 0.85 | 0.136 |
|  |  | 69744886 | rs3798984 | A/G | 0.103 | 0.118 | 0.982 | 0.158 |
|  |  | 69747288 | rs12665384 | A/G | 0.111 | 0.098 | 0.556 | 0.207 |
|  |  | 69755259 | rs3798992 | T/G | 0.452 | 0.457 | 0.992 | 0.75 |
|  |  | 69755313 | rs3798993 | A/G | 0.452 | 0.456 | 0.959 | 0.777 |
|  |  | 69759137 | rs9454667 | G/A | 0.311 | 0.298 | 0.918 | 0.422 |
|  |  | 69762634 | rs3798996 | G/A | 0.099 | 0.088 | 0.604 | 0.24 |
|  |  | 69765639 | rs6908180 | G/A | 0.1 | 0.087 | 0.522 | 0.194 |
|  |  | 69767438 | rs1415030 | A/G | 0.242 | 0.266 | 0.967 | 0.102 |
|  |  | 69769778 | rs13191563 | G/A | 0.246 | 0.233 | 0.859 | 0.361 |
|  |  | 69771668 | rs3798999 | G/A | 0.42 | 0.442 | 0.888 | 0.186 |
|  |  | 69774879 | rs12213405 | C/T | 0.468 | 0.463 | 0.43 | 0.738 |
|  |  | 69779766 | rs3799007 | T/C | 0.063 | 0.053 | 0.57 | 0.187 |
|  |  | 69782303 | rs2785575 | G/T | 0.132 | 0.139 | 0.543 | 0.542 |
|  |  | 69785173 | rs10485430 | C/T | 0.164 | 0.155 | 0.703 | 0.455 |
|  |  | 69790141 | rs1336653 | A/G | 0.145 | 0.143 | 0.502 | 0.878 |
|  |  | 69802444 | rs2225803 | G/A | 0.208 | 0.193 | 0.933 | 0.285 |
|  |  | 69802775 | rs2210867 | C/T | 0.469 | 0.461 | 0.544 | 0.614 |
|  |  | 69803168 | rs7756040 | G/A | 0.469 | 0.461 | 0.535 | 0.616 |
|  |  | 69805321 | rs11962923 | T/G | 0.161 | 0.155 | 0.849 | 0.602 |
|  |  | 69809791 | rs3799019 | C/T | 0.11 | 0.094 | 0.325 | 0.134 |
|  |  | 69812831 | rs6904267 | G/T | 0.47 | 0.46 | 0.58 | 0.558 |
|  |  | 69823230 | rs634371 | G/A | 0.48 | 0.471 | 0.586 | 0.58 |
|  |  | 69824308 | rs9446089 | G/A | 0.264 | 0.263 | 0.381 | 0.982 |
|  |  | 69827885 | rs10485428 | T/C | 0.07 | 0.077 | 0.984 | 0.446 |
|  |  | 69837930 | rs526898 | C/A | 0.479 | 0.466 | 0.788 | 0.418 |
|  |  | 69850758 | rs3823070 | T/G | 0.082 | 0.065 | 0.273 | 0.061 |
|  |  | 69850800 | rs11964764 | G/T | 0.193 | 0.191 | 0.954 | 0.845 |
|  |  | 69854719 | rs994380 | C/T | 0.297 | 0.29 | 0.785 | 0.665 |
|  |  | 69859223 | rs10455681 | A/G | 0.152 | 0.151 | 0.852 | 0.909 |
|  |  | 69860183 | rs6913677 | G/A | 0.346 | 0.339 | 0.691 | 0.659 |
|  |  | 69860895 | rs2170509 | A/G | 0.295 | 0.29 | 0.883 | 0.728 |
|  |  | 69862706 | rs3799033 | T/G | 0.02 | 0.024 | 0.891 | 0.362 |
|  |  | 69873172 | rs10485427 | A/G | 0.262 | 0.259 | 0.596 | 0.87 |
|  |  | 69875314 | rs9363983 | G/T | 0.201 | 0.203 | 0.536 | 0.881 |
|  |  | 69883228 | rs3799039 | A/G | 0.439 | 0.45 | 0.409 | 0.539 |
|  |  | 69893100 | rs9294819 | C/T | 0.238 | 0.246 | 0.122 | 0.591 |
|  |  | 69896830 | rs9360376 | A/G | 0.341 | 0.333 | 0.754 | 0.627 |
|  |  | 69901489 | rs1482326 | G/A | 0.294 | 0.304 | 0.793 | 0.478 |
|  |  | 69905924 | rs11759629 | C/T | 0.168 | 0.163 | 0.654 | 0.645 |
|  |  | 69908537 | rs1912988 | C/T | 0.171 | 0.171 | 0.898 | 0.97 |
|  |  | 69908591 | rs1912987 | G/T | 0.171 | 0.17 | 0.989 | 0.892 |
|  |  | 69922362 | rs3799046 | C/A | 0.347 | 0.335 | 0.269 | 0.434 |
|  |  | 69956823 | rs779484 | T/C | 0.278 | 0.287 | 0.801 | 0.541 |
|  |  | 69959797 | rs779482 | A/C | 0.346 | 0.332 | 0.288 | 0.399 |
|  |  | 69961822 | rs16900558 | A/G | 0.123 | 0.124 | 0.719 | 0.924 |
|  |  | 69968691 | rs779480 | G/A | 0.344 | 0.332 | 0.32 | 0.464 |
|  |  | 69981003 | rs779473 | C/T | 0.158 | 0.166 | 0.823 | 0.519 |
|  |  | 69984990 | rs1877427 | A/G | 0.183 | 0.166 | 0.205 | 0.212 |
|  |  | 69988140 | rs779467 | C/T | 0.092 | 0.101 | 0.31 | 0.385 |
|  |  | 69991060 | rs1619465 | C/A | 0.469 | 0.454 | 0.338 | 0.379 |
|  |  | 69993900 | rs971564 | C/T | 0.183 | 0.166 | 0.19 | 0.196 |
|  |  | 70000953 | rs1512229 | C/T | 0.183 | 0.166 | 0.166 | 0.183 |
|  |  | 70005192 | rs779460 | A/G | 0.278 | 0.29 | 0.96 | 0.402 |
|  |  | 70010435 | rs779456 | C/T | 0.159 | 0.166 | 0.851 | 0.558 |
|  |  | 70010707 | rs10485254 | G/A | 0.179 | 0.164 | 0.259 | 0.253 |
|  |  | 70016039 | rs12209170 | G/A | 0.213 | 0.215 | 0.439 | 0.885 |
|  |  | 70019740 | rs779453 | G/A | 0.066 | 0.074 | 0.932 | 0.361 |
|  |  | 70020740 | rs3799070 | A/C | 0.211 | 0.213 | 0.469 | 0.831 |
|  |  | 70021017 | rs779452 | C/T | 0.461 | 0.45 | 0.466 | 0.513 |
|  |  | 70027899 | rs9454720 | A/G | 0.16 | 0.167 | 0.858 | 0.592 |
|  |  | 70036710 | rs6919398 | G/A | 0.065 | 0.065 | 0.404 | 0.966 |
|  |  | 70051082 | rs10485252 | T/C | 0.375 | 0.373 | 0.658 | 0.904 |
|  |  | 70060110 | rs9454727 | A/G | 0.283 | 0.281 | 0.703 | 0.859 |
|  |  | 70060330 | rs1321980 | G/A | 0.123 | 0.113 | 0.265 | 0.328 |
|  |  | 70064171 | rs3799081 | C/A | 0.378 | 0.383 | 0.768 | 0.779 |
|  |  | 70064271 | rs1885331 | T/G | 0.225 | 0.236 | 0.886 | 0.459 |
|  |  | 70066946 | rs13191298 | A/G | 0.147 | 0.137 | 0.372 | 0.383 |
|  |  | 70071329 | rs1321981 | T/G | 0.396 | 0.4 | 0.736 | 0.786 |
|  |  | 70074971 | rs13195278 | A/G | 0.089 | 0.098 | 0.289 | 0.348 |
|  |  | 70084140 | rs1283464 | A/G | 0.089 | 0.098 | 0.262 | 0.316 |
|  |  | 70086110 | rs10485251 | A/C | 0.198 | 0.191 | 0.707 | 0.627 |
|  |  | 70086701 | rs1290329 | C/A | 0.372 | 0.378 | 0.551 | 0.67 |
|  |  | 70098643 | rs1283471 | G/A | 0.017 | 0.019 | 0.901 | 0.513 |
|  |  | 70098766 | rs17748715 | G/A | 0.162 | 0.177 | 0.438 | 0.236 |
|  |  | 70116946 | rs1296346 | T/C | 0.167 | 0.183 | 0.241 | 0.205 |
|  |  | 70119646 | rs1033758 | C/A | 0.026 | 0.029 | 0.869 | 0.55 |
|  |  | 70123341 | rs6912952 | T/C | 0.207 | 0.196 | 0.734 | 0.447 |
|  |  | 70133454 | rs1410700 | G/A | 0.164 | 0.178 | 0.35 | 0.234 |
|  |  | 70137233 | rs3799094 | G/T | 0.158 | 0.15 | 0.817 | 0.499 |
|  |  | 70152411 | rs1952435 | T/C | 0.341 | 0.344 | 0.41 | 0.846 |
|  |  | 70154309 | rs1328735 | G/A | 0.432 | 0.431 | 0.445 | 0.935 |
|  |  | 70160744 | rs9346273 | C/T | 0.17 | 0.157 | 0.897 | 0.311 |
|  |  | 70161403 | rs1328734 | T/G | 0.491 | 0.499 | 0.785 | 0.516 |
|  |  | 70165569 | rs1328729 | C/T | 0.203 | 0.196 | 0.766 | 0.619 |
|  |  | 70171553 | rs6927885 | C/A | 0.478 | 0.465 | 0.622 | 0.444 |
|  |  | 70175562 | rs1328726 | A/G | 0.095 | 0.098 | 0.527 | 0.816 |
|  |  | 70178582 | rs9354833 | C/T | 0.488 | 0.476 | 0.611 | 0.438 |
|  |  | 70184425 | rs1928055 | A/G | 0.048 | 0.046 | 0.56 | 0.855 |
|  |  | 70192792 | rs4707025 | T/C | 0.383 | 0.368 | 0.908 | 0.373 |
|  |  | 70193166 | rs7762967 | C/T | 0.048 | 0.046 | 0.544 | 0.819 |
|  |  | 70195900 | rs2026018 | A/G | 0.07 | 0.07 | 0.662 | 0.942 |
|  |  | 70201287 | rs12211885 | T/C | 0.048 | 0.047 | 0.596 | 0.876 |
|  |  | 70204802 | rs1360025 | G/A | 0.249 | 0.277 | 0.283 | 0.055 |
|  |  | 70225903 | rs17694107 | T/C | 0.151 | 0.127 | 0.236 | 0.047 |
|  |  | 70233637 | rs2094596 | G/A | 0.079 | 0.079 | 0.669 | 0.957 |
|  |  | 70240308 | rs1887845 | A/G | 0.263 | 0.254 | 0.82 | 0.537 |
|  |  | 70242690 | rs1410692 | T/C | 0.373 | 0.356 | 0.83 | 0.31 |
|  | STXBP5 ^(D1)^ | 147485173 | rs9497707 | T/C | 0.05 | 0.04 | 0.419 | 0.192 |
|  |  | 147499177 | rs9322091 | T/C | 0.011 | 0.011 | 0.205 | 0.98 |
|  |  | 147513423 | rs2256216 | G/A | 0.433 | 0.423 | 0.52 | 0.554 |
|  |  | 147532091 | rs1660690 | C/T | 0.436 | 0.48 | 0.027 | 7.87 10^-3^ |
|  |  | 147534435 | rs693573 | T/C | 0.432 | 0.474 | 0.04 | 0.01 |
|  |  | 147542177 | rs694625 | A/G | 0.499 | 0.461 | 0.057 | 0.016 |
|  |  | 147542474 | rs2818867 | A/G | 0.474 | 0.447 | 0.154 | 0.103 |
|  |  | 147552159 | rs1630961 | C/A | 0.413 | 0.396 | 0.522 | 0.307 |
|  |  | 147580858 | rs1765028 | A/G | 0.443 | 0.435 | 0.645 | 0.652 |
|  |  | 147611932 | rs620715 | G/T | 0.446 | 0.45 | 0.785 | 0.826 |
|  |  | 147613528 | rs580657 | A/G | 0.447 | 0.45 | 0.796 | 0.852 |
|  |  | 147619339 | rs10499247 | C/A | 0.04 | 0.037 | 0.601 | 0.698 |
|  |  | 147632715 | rs597631 | G/A | 0.448 | 0.452 | 0.776 | 0.827 |
|  |  | 147662094 | rs2850177 | A/C | 0.41 | 0.416 | 0.632 | 0.685 |
|  |  | 147677106 | rs1039084 | G/A | 0.447 | 0.452 | 0.758 | 0.754 |
|  |  | 147686198 | rs4896904 | C/T | 0.406 | 0.413 | 0.661 | 0.703 |
|  |  | 147722052 | rs9390459 | G/A | 0.423 | 0.429 | 0.783 | 0.696 |
|  |  | 147727173 | rs6924763 | G/A | 0.158 | 0.158 | 0.682 | 0.974 |
|  |  | 147727967 | rs9497759 | A/G | 0.386 | 0.399 | 0.522 | 0.448 |
|  |  | 147750873 | rs7739314 | C/A | 0.455 | 0.459 | 0.915 | 0.824 |
|  | PLG ^(B)^ | 161027008 | rs9457997 | G/A | 0.148 | 0.16 | 0.45 | 0.309 |
|  |  | 161028526 | rs6935921 | T/C | 0.272 | 0.267 | 0.639 | 0.754 |
|  |  | 161029892 | rs9457999 | C/T | 0.229 | 0.234 | 0.529 | 0.713 |
|  |  | 161038567 | rs9458005 | A/G | 0.219 | 0.23 | 0.384 | 0.437 |
|  |  | 161056284 | rs9295131 | A/G | 0.304 | 0.291 | 0.489 | 0.384 |
|  |  | 161057980 | rs783147 | G/A | 0.467 | 0.453 | 0.169 | 0.411 |
|  |  | 161058907 | rs3823055 | G/A | 0.272 | 0.275 | 0.697 | 0.829 |
|  |  | 161059470 | rs1130656 | C/T | 0.389 | 0.41 | 0.131 | 0.192 |
|  |  | 161059847 | rs13231 | A/G | 0.308 | 0.299 | 0.324 | 0.557 |
|  |  | 161064719 | rs1853025 | G/A | 0.031 | 0.015 | 0.376 | 1.62 10^-3^ |
|  |  | 161072230 | rs4252125 | G/A | 0.309 | 0.301 | 0.443 | 0.624 |
|  |  | 161072439 | rs783145 | A/G | 0.469 | 0.476 | 0.924 | 0.676 |
|  |  | 161073980 | rs813641 | G/A | 0.159 | 0.152 | 0.475 | 0.596 |
|  |  | 161082396 | rs4252170 | T/C | 0.088 | 0.093 | 0.602 | 0.569 |
|  |  | 161097227 | rs783166 | G/A | 0.083 | 0.085 | 0.295 | 0.855 |
|  |  | 161106879 | rs7450979 | G/T | 0.342 | 0.338 | 0.674 | 0.803 |
|  |  | 161107070 | rs1406891 | C/T | 0.456 | 0.466 | 0.843 | 0.562 |
|  |  | 161110189 | rs1247558 | G/A | 0.477 | 0.482 | 0.73 | 0.732 |
|  |  | 161112179 | rs1652500 | T/C | 0.181 | 0.195 | 0.376 | 0.284 |
|  |  | 161116790 | rs1782627 | A/G | 0.464 | 0.459 | 0.783 | 0.722 |
|  |  | 161118073 | rs1652483 | G/T | 0.179 | 0.178 | 0.903 | 0.927 |
|  |  | 161121950 | rs13199812 | T/C | 0.357 | 0.348 | 0.593 | 0.575 |
|  |  | 161136598 | rs2064712 | G/A | 0.147 | 0.148 | 0.945 | 0.916 |
|  |  | 161138641 | rs1247571 | G/A | 0.149 | 0.164 | 0.464 | 0.206 |
|  |  | 161145293 | rs1590185 | T/C | 0.339 | 0.335 | 0.46 | 0.789 |
|  |  | 161152761 | rs783157 | T/C | 0.17 | 0.18 | 0.343 | 0.419 |
|  |  | 161153171 | rs13202192 | C/T | 0.349 | 0.336 | 0.305 | 0.426 |
|  |  | 161155088 | rs2565721 | G/A | 0.47 | 0.473 | 0.854 | 0.832 |
|  |  | 161155700 | rs9458041 | C/T | 0.35 | 0.335 | 0.217 | 0.337 |
|  |  | 161160831 | rs12527129 | A/C | 0.018 | 0.016 | 0.7 | 0.545 |
|  |  | 161180520 | rs2489952 | A/G | 0.175 | 0.183 | 0.354 | 0.562 |
|  |  | 161187325 | rs9355841 | G/A | 0.444 | 0.447 | 0.801 | 0.839 |
| 7 | BAZ1B ^(D3)^ | 72494205 | rs2240466 | G/A | 0.099 | 0.086 | 0.048 | 0.154 |
|  |  | 72499718 | rs2237280 | C/T | 0.034 | 0.027 | 0.42 | 0.21 |
|  |  | 72523746 | rs6976930 | G/A | 0.165 | 0.149 | 0.038 | 0.17 |
|  |  | 72529690 | rs2074754 | C/T | 0.448 | 0.474 | 0.68 | 0.119 |
|  |  | 72534486 | rs17400042 | A/G | 0.031 | 0.025 | 0.494 | 0.292 |
|  | SERPINE1 ^(B)^ | 100541637 | rs12669411 | A/G | 0.133 | 0.131 | 0.876 | 0.89 |
|  |  | 100553323 | rs6950982 | A/G | 0.206 | 0.207 | 0.816 | 0.962 |
|  |  | 100556258 | rs2227631 | A/G | 0.418 | 0.417 | 0.706 | 0.939 |
|  |  | 100562406 | rs2227672 | G/T | 0.126 | 0.135 | 0.408 | 0.422 |
|  |  | 100563651 | rs2227684 | G/A | 0.423 | 0.424 | 0.543 | 0.911 |
|  |  | 100568165 | rs7242 | T/G | 0.422 | 0.425 | 0.476 | 0.856 |
|  |  | 100568335 | rs1050813 | G/A | 0.22 | 0.216 | 0.561 | 0.757 |
| 8 | SCARA5^(D1)^ | 27767592 | rs4732776 | C/T | 0.34 | 0.331 | 0.295 | 0.564 |
|  |  | 27771035 | rs1870587 | T/C | 0.107 | 0.096 | 0.056 | 0.286 |
|  |  | 27772227 | rs7823922 | T/C | 0.341 | 0.331 | 0.281 | 0.549 |
|  |  | 27774219 | rs4537259 | G/A | 0.106 | 0.095 | 0.047 | 0.265 |
|  |  | 27776290 | rs4403364 | T/G | 0.238 | 0.245 | 0.594 | 0.621 |
|  |  | 27776743 | rs898460 | A/G | 0.347 | 0.34 | 0.438 | 0.648 |
|  |  | 27785731 | rs898464 | G/A | 0.069 | 0.054 | 0.014 | 0.06 |
|  |  | 27790412 | rs939717 | G/A | 0.39 | 0.415 | 0.246 | 0.118 |
|  |  | 27796759 | rs10091985 | C/A | 0.465 | 0.488 | 0.246 | 0.179 |
|  |  | 27797474 | rs4545047 | T/C | 0.224 | 0.201 | 0.178 | 0.086 |
|  |  | 27800283 | rs10090871 | A/C | 0.345 | 0.346 | 0.844 | 0.95 |
|  |  | 27801124 | rs1879678 | A/G | 0.154 | 0.14 | 0.645 | 0.24 |
|  |  | 27805263 | rs2726959 | G/T | 0.193 | 0.195 | 0.314 | 0.897 |
|  |  | 27805313 | rs2726960 | T/C | 0.349 | 0.342 | 0.234 | 0.65 |
|  |  | 27805759 | rs4545048 | T/C | 0.103 | 0.106 | 0.384 | 0.804 |
|  |  | 27807298 | rs2685376 | C/T | 0.058 | 0.051 | 0.633 | 0.349 |
|  |  | 27809607 | rs7817722 | A/G | 0.136 | 0.143 | 0.17 | 0.499 |
|  |  | 27810746 | rs2726972 | T/C | 0.079 | 0.077 | 0.609 | 0.824 |
|  |  | 27814295 | rs7002829 | C/T | 0.021 | 0.029 | 0.014 | 0.093 |
|  |  | 27815045 | rs2685372 | C/T | 0.381 | 0.367 | 0.934 | 0.361 |
|  |  | 27816394 | rs6999364 | T/C | 0.371 | 0.355 | 0.296 | 0.328 |
|  |  | 27819048 | rs4515501 | G/A | 0.277 | 0.265 | 0.579 | 0.396 |
|  |  | 27822394 | rs2685358 | C/T | 0.36 | 0.345 | 0.352 | 0.342 |
|  |  | 27822985 | rs17058204 | G/A | 0.08 | 0.078 | 0.432 | 0.877 |
|  |  | 27831036 | rs2685325 | T/C | 0.246 | 0.243 | 0.321 | 0.831 |
|  |  | 27834761 | rs2685320 | T/C | 0.339 | 0.339 | 0.11 | 0.98 |
|  |  | 27837517 | rs11136019 | T/C | 0.351 | 0.344 | 0.04 | 0.643 |
|  |  | 27851730 | rs2726958 | C/A | 0.066 | 0.069 | 0.513 | 0.652 |
|  |  | 27852970 | rs4472469 | G/A | 0.367 | 0.361 | 0.071 | 0.7 |
|  |  | 27859518 | rs4276643 | T/C | 0.3 | 0.296 | 0.161 | 0.816 |
|  |  | 27864913 | rs10866867 | G/T | 0.256 | 0.254 | 0.194 | 0.895 |
|  |  | 27868026 | rs884829 | T/C | 0.256 | 0.256 | 0.341 | 0.956 |
|  |  | 27870402 | rs9644133 | C/T | 0.174 | 0.186 | 0.333 | 0.363 |
|  |  | 27871605 | rs17392666 | C/T | 0.054 | 0.05 | 0.868 | 0.554 |
|  |  | 27873403 | rs2726943 | C/T | 0.196 | 0.199 | 0.34 | 0.827 |
|  |  | 27875668 | rs2726942 | C/T | 0.449 | 0.468 | 0.138 | 0.275 |
|  |  | 27877751 | rs2685403 | T/C | 0.206 | 0.206 | 0.304 | 0.951 |
|  |  | 27880206 | rs939705 | A/C | 0.452 | 0.469 | 0.138 | 0.317 |
|  |  | 27880527 | rs2726938 | T/G | 0.452 | 0.469 | 0.159 | 0.307 |
|  |  | 27881479 | rs17393091 | T/G | 0.27 | 0.27 | 0.864 | 0.98 |
|  |  | 27881693 | rs11779320 | C/T | 0.154 | 0.17 | 0.3 | 0.179 |
|  |  | 27882479 | rs17058386 | G/T | 0.038 | 0.039 | 0.944 | 0.842 |
|  |  | 27885170 | rs4732617 | A/G | 0.306 | 0.291 | 0.452 | 0.302 |
|  |  | 27892336 | rs1001866 | C/T | 0.383 | 0.391 | 0.625 | 0.612 |
|  |  | 27896535 | rs11780951 | G/A | 0.444 | 0.426 | 0.618 | 0.27 |
|  |  | 27904145 | rs2685391 | G/A | 0.294 | 0.294 | 0.225 | 0.986 |
|  |  | 27909278 | rs2726961 | T/C | 0.245 | 0.242 | 0.198 | 0.836 |
|  |  | 27912250 | rs13268059 | G/A | 0.351 | 0.339 | 0.699 | 0.464 |
|  | PLAT ^(B)^ | 42151183 | rs4581040 | A/G | 0.311 | 0.314 | 0.878 | 0.864 |
|  |  | 42155734 | rs1136159 | A/G | 0.104 | 0.111 | 0.152 | 0.539 |
|  |  | 42164812 | rs2070713 | C/T | 0.409 | 0.401 | 0.479 | 0.591 |
|  |  | 42196227 | rs7002654 | A/G | 0.049 | 0.052 | 0.272 | 0.727 |
|  |  | 42205629 | rs7820274 | C/T | 0.323 | 0.322 | 0.63 | 0.938 |
|  |  | 42206733 | rs10094577 | T/C | 0.05 | 0.053 | 0.201 | 0.63 |
| 10 | PLAU  ^(B)^ | 75343107 | rs2227564 | C/T | 0.206 | 0.199 | 0.023 | 0.633 |
|  |  | 75353799 | rs16931023 | G/A | 0.15 | 0.164 | 0.104 | 0.235 |
|  |  | 75363701 | rs2633321 | A/G | 0.357 | 0.378 | 0.683 | 0.184 |
|  |  | 75363938 | rs2633322 | C/T | 0.207 | 0.208 | 0.109 | 0.972 |
|  |  | 75371861 | rs4746158 | A/G | 0.282 | 0.291 | 0.387 | 0.538 |
|  | HABP2 ^(D5)^ | 115267972 | rs11196356 | T/C | 0.427 | 0.405 | 0.945 | 0.195 |
|  |  | 115271847 | rs2419831 | A/G | 0.253 | 0.227 | 0.322 | 0.068 |
|  |  | 115275298 | rs11592949 | T/G | 0.024 | 0.02 | 0.592 | 0.405 |
|  |  | 115277192 | rs1570269 | G/T | 0.103 | 0.087 | 0.774 | 0.093 |
|  |  | 115279365 | rs4261225 | G/T | 0.082 | 0.067 | 0.644 | 0.095 |
|  |  | 115282428 | rs11196361 | C/T | 0.188 | 0.187 | 0.43 | 0.977 |
|  |  | 115284695 | rs7088233 | C/T | 0.141 | 0.133 | 0.446 | 0.49 |
|  |  | 115288290 | rs11196366 | C/T | 0.142 | 0.137 | 0.438 | 0.678 |
|  |  | 115292566 | rs11595042 | G/A | 0.022 | 0.018 | 0.465 | 0.352 |
|  |  | 115298446 | rs1853204 | A/G | 0.253 | 0.225 | 0.224 | 0.054 |
|  |  | 115300088 | rs2000278 | C/A | 0.373 | 0.359 | 0.458 | 0.389 |
|  |  | 115304488 | rs10509980 | A/C | 0.397 | 0.382 | 0.568 | 0.356 |
|  |  | 115306715 | rs10509981 | C/A | 0.316 | 0.337 | 0.898 | 0.168 |
|  |  | 115306802 | rs4918842 | T/C | 0.143 | 0.14 | 0.718 | 0.769 |
|  |  | 115309701 | rs4918844 | T/C | 0.301 | 0.286 | 0.14 | 0.301 |
|  |  | 115311814 | rs11575634 | C/T | 0.151 | 0.142 | 0.657 | 0.456 |
|  |  | 115312143 | rs4545483 | A/C | 0.387 | 0.407 | 0.926 | 0.218 |
|  |  | 115314348 | rs11196379 | A/G | 0.258 | 0.251 | 0.597 | 0.605 |
|  |  | 115314489 | rs7088038 | A/G | 0.47 | 0.489 | 0.662 | 0.254 |
|  |  | 115315435 | rs4918851 | C/A | 0.258 | 0.25 | 0.558 | 0.612 |
|  |  | 115317074 | rs11575668 | A/G | 0.105 | 0.101 | 0.68 | 0.707 |
|  |  | 115319336 | rs3850691 | A/G | 0.21 | 0.198 | 0.959 | 0.366 |
|  |  | 115319876 | rs11575674 | G/A | 0.01 | 0.015 | 0.472 | 0.175 |
|  |  | 115320120 | rs4918853 | C/T | 0.185 | 0.183 | 0.252 | 0.856 |
|  |  | 115320711 | rs10885476 | G/A | 0.178 | 0.168 | 0.526 | 0.419 |
|  |  | 115321608 | rs911707 | C/A | 0.182 | 0.176 | 0.496 | 0.634 |
|  |  | 115322088 | rs2419840 | A/C | 0.416 | 0.402 | 0.043 | 0.368 |
|  |  | 115322403 | rs10885477 | C/T | 0.071 | 0.067 | 0.498 | 0.619 |
|  |  | 115324114 | rs3740530 | T/C | 0.397 | 0.385 | 0.338 | 0.47 |
|  |  | 115324626 | rs11196383 | C/T | 0.07 | 0.065 | 0.416 | 0.54 |
|  |  | 115326357 | rs7923349 | G/T | 0.254 | 0.247 | 0.561 | 0.614 |
|  |  | 115326748 | rs2302374 | A/C | 0.189 | 0.216 | 0.249 | 0.051 |
|  |  | 115327383 | rs1885437 | G/A | 0.398 | 0.414 | 0.335 | 0.335 |
|  |  | 115330482 | rs2286742 | A/G | 0.465 | 0.443 | 0.631 | 0.192 |
|  |  | 115333277 | rs2302373 | T/C | 0.394 | 0.384 | 0.606 | 0.545 |
|  |  | 115333690 | rs911704 | G/A | 0.19 | 0.198 | 0.481 | 0.561 |
|  |  | 115334779 | rs932652 | G/T | 0.416 | 0.418 | 0.282 | 0.888 |
|  |  | 115337349 | rs932650 | T/C | 0.348 | 0.352 | 0.526 | 0.762 |
| 11 | F2 ^(A)^ | 46690960 | rs11038977 | C/T | 0.095 | 0.093 | 0.255 | 0.809 |
|  |  | 46701579 | rs5896 | C/T | 0.095 | 0.091 | 0.224 | 0.741 |
|  |  | 46702437 | rs3136456 | C/A | 0.06 | 0.078 | 8.91 10^-3^ | 0.034 |
| 12 | VWF^(A)^ | 5931221 | rs2270151 | G/A | 0.167 | 0.186 | 0.123 | 0.138 |
|  |  | 5931752 | rs2286646 | A/G | 0.236 | 0.239 | 0.811 | 0.843 |
|  |  | 5933403 | rs12317523 | C/T | 0.297 | 0.29 | 0.759 | 0.627 |
|  |  | 5934166 | rs723188 | G/A | 0.205 | 0.179 | 0.152 | 0.044 |
|  |  | 5941100 | rs2363309 | G/A | 0.494 | 0.467 | 0.475 | 0.095 |
|  |  | 5941777 | rs12368267 | T/C | 0.135 | 0.124 | 0.71 | 0.363 |
|  |  | 5944277 | rs11063961 | A/G | 0.243 | 0.232 | 0.797 | 0.413 |
|  |  | 5949319 | rs12300917 | C/T | 0.233 | 0.224 | 0.757 | 0.542 |
|  |  | 5952085 | rs917857 | G/A | 0.466 | 0.457 | 0.905 | 0.603 |
|  |  | 5952374 | rs917859 | G/A | 0.298 | 0.305 | 0.594 | 0.63 |
|  |  | 5953825 | rs1159993 | T/G | 0.021 | 0.025 | 0.573 | 0.436 |
|  |  | 5954584 | rs4764521 | G/A | 0.168 | 0.168 | 0.438 | 0.974 |
|  |  | 5956526 | rs216856 | C/T | 0.351 | 0.34 | 0.341 | 0.487 |
|  |  | 5961261 | rs216867 | G/A | 0.124 | 0.112 | 0.496 | 0.249 |
|  |  | 5963801 | rs216872 | T/C | 0.284 | 0.285 | 0.659 | 0.963 |
|  |  | 5964187 | rs2058473 | A/G | 0.363 | 0.371 | 0.301 | 0.624 |
|  |  | 5964213 | rs2070887 | T/C | 0.077 | 0.085 | 0.362 | 0.37 |
|  |  | 5967918 | rs216883 | C/T | 0.221 | 0.228 | 0.613 | 0.612 |
|  |  | 5969714 | rs216889 | C/T | 0.486 | 0.485 | 0.447 | 0.95 |
|  |  | 5971057 | rs216896 | T/C | 0.49 | 0.486 | 0.461 | 0.827 |
|  |  | 5973168 | rs10849376 | G/A | 0.077 | 0.085 | 0.343 | 0.379 |
|  |  | 5974105 | rs17491334 | G/A | 0.147 | 0.142 | 0.16 | 0.683 |
|  |  | 5976279 | rs216904 | T/C | 0.362 | 0.367 | 0.959 | 0.722 |
|  |  | 5985535 | rs216811 | G/A | 0.287 | 0.302 | 0.638 | 0.312 |
|  |  | 5999245 | rs216312 | C/T | 0.424 | 0.427 | 0.184 | 0.854 |
|  |  | 5999525 | rs216313 | A/G | 0.062 | 0.061 | 0.462 | 0.892 |
|  |  | 6006895 | rs11611917 | G/A | 0.259 | 0.24 | 0.013 | 0.185 |
|  |  | 6007717 | rs11610629 | A/C | 0.257 | 0.24 | 0.014 | 0.229 |
|  |  | 6014245 | rs216321 | C/T | 0.072 | 0.077 | 0.533 | 0.559 |
|  |  | 6014399 | rs12810426 | G/A | 0.232 | 0.221 | 0.03 | 0.428 |
|  |  | 6017872 | rs216334 | T/C | 0.328 | 0.316 | 0.036 | 0.44 |
|  |  | 6018266 | rs216335 | C/T | 0.073 | 0.078 | 0.648 | 0.598 |
|  |  | 6018587 | rs216338 | G/A | 0.37 | 0.36 | 0.073 | 0.506 |
|  |  | 6020060 | rs216340 | G/A | 0.18 | 0.191 | 0.137 | 0.402 |
|  |  | 6023795 | rs1063856 | T/C | 0.378 | 0.35 | 1.79 10^-3^ | 0.08 |
|  |  | 6023920 | rs216293 | T/G | 0.45 | 0.429 | 7.57 10^-3^ | 0.215 |
|  |  | 6026738 | rs216298 | T/C | 0.072 | 0.079 | 0.533 | 0.423 |
|  |  | 6033113 | rs2283333 | C/T | 0.089 | 0.097 | 0.42 | 0.422 |
|  |  | 6039288 | rs980130 | C/T | 0.317 | 0.305 | 0.114 | 0.412 |
|  |  | 6039459 | rs980131 | C/T | 0.401 | 0.383 | 9.09 10^-3^ | 0.28 |
|  |  | 6039584 | rs7139057 | T/G | 0.207 | 0.189 | 0.021 | 0.171 |
|  |  | 6039994 | rs4764482 | C/T | 0.479 | 0.466 | 0.111 | 0.422 |
|  |  | 6040747 | rs12319392 | G/T | 0.085 | 0.089 | 0.156 | 0.708 |
|  |  | 6042463 | rs1800378 | C/T | 0.341 | 0.327 | 0.102 | 0.398 |
|  |  | 6044990 | rs2283332 | G/A | 0.091 | 0.073 | 9.05 10^-3^ | 0.06 |
|  |  | 6045840 | rs7955850 | T/G | 0.104 | 0.088 | 0.04 | 0.102 |
|  |  | 6053442 | rs3782711 | T/C | 0.155 | 0.131 | 0.076 | 0.042 |
|  |  | 6057926 | rs2238104 | G/T | 0.453 | 0.47 | 0.47 | 0.302 |
|  |  | 6066444 | rs2239144 | C/A | 0.157 | 0.137 | 0.447 | 0.104 |
|  |  | 6070704 | rs2239140 | C/T | 0.487 | 0.499 | 0.317 | 0.467 |
|  |  | 6072310 | rs11064024 | A/G | 0.346 | 0.345 | 0.387 | 0.992 |
|  |  | 6073079 | rs11836843 | A/G | 0.078 | 0.073 | 0.314 | 0.615 |
|  |  | 6085895 | rs7306706 | A/G | 0.452 | 0.446 | 0.793 | 0.745 |
|  |  | 6108740 | rs10849387 | T/C | 0.368 | 0.364 | 0.634 | 0.786 |
|  |  | 6113877 | rs10774398 | C/T | 0.39 | 0.385 | 0.476 | 0.743 |
|  |  | 6125686 | rs6489695 | G/A | 0.343 | 0.337 | 0.571 | 0.706 |
|  |  | 6134080 | rs11064058 | A/C | 0.379 | 0.377 | 0.362 | 0.931 |
|  |  | 6141364 | rs10849399 | C/T | 0.375 | 0.38 | 0.313 | 0.756 |
|  | STAB2^(D1)^ | 102464883 | rs11111642 | C/T | 0.027 | 0.024 | 0.785 | 0.579 |
|  |  | 102466587 | rs4405407 | A/G | 0.159 | 0.175 | 0.131 | 0.196 |
|  |  | 102466844 | rs4540923 | C/T | 0.281 | 0.312 | 0.247 | 0.034 |
|  |  | 102475259 | rs4131522 | C/T | 0.202 | 0.167 | 0.248 | 7.50 10^-3^ |
|  |  | 102477187 | rs10861038 | C/A | 0.072 | 0.067 | 0.964 | 0.559 |
|  |  | 102483314 | rs10778263 | C/A | 0.409 | 0.42 | 0.064 | 0.486 |
|  |  | 102491858 | rs1346412 | A/G | 0.459 | 0.462 | 0.145 | 0.867 |
|  |  | 102498385 | rs10454504 | G/A | 0.091 | 0.081 | 0.154 | 0.29 |
|  |  | 102499779 | rs1593812 | A/G | 0.167 | 0.135 | 1.39 10^-3^ | 0.446 |
|  |  | 102499895 | rs10745975 | C/T | 0.092 | 0.082 | 0.142 | 0.284 |
|  |  | 102501396 | rs1582880 | A/G | 0.487 | 0.496 | 0.471 | 0.608 |
|  |  | 102511875 | rs10507167 | A/G | 0.052 | 0.046 | 0.817 | 0.471 |
|  |  | 102512025 | rs1582881 | G/A | 0.482 | 0.494 | 0.714 | 0.452 |
|  |  | 102512247 | rs703596 | C/A | 0.171 | 0.209 | 7.54 10^-3^ | 3.16 10^-3^ |
|  |  | 102517696 | rs1593806 | A/G | 0.272 | 0.227 | 1.67 10^-3^ | 0.047 |
|  |  | 102535143 | rs703611 | T/G | 0.201 | 0.234 | 0.041 | 0.015 |
|  |  | 102544245 | rs7133148 | T/C | 0.311 | 0.346 | 0.054 | 0.023 |
|  |  | 102544362 | rs703616 | C/T | 0.485 | 0.44 | 0.012 | 5.07 10^-3^ |
|  |  | 102547434 | rs1018034 | C/A | 0.169 | 0.162 | 0.434 | 0.581 |
|  |  | 102549115 | rs11111687 | T/C | 0.088 | 0.077 | 0.031 | 0.263 |
|  |  | 102551715 | rs1677979 | C/T | 0.322 | 0.29 | 0.204 | 0.038 |
|  |  | 102551947 | rs1993919 | A/G | 0.131 | 0.11 | 0.013 | 0.049 |
|  |  | 102553592 | rs11111689 | C/T | 0.138 | 0.125 | 0.053 | 0.255 |
|  |  | 102555541 | rs10861063 | A/G | 0.249 | 0.23 | 0.597 | 0.184 |
|  |  | 102563104 | rs10861067 | A/G | 0.285 | 0.272 | 0.428 | 0.403 |
|  |  | 102566866 | rs697199 | C/T | 0.269 | 0.253 | 0.907 | 0.275 |
|  |  | 102569808 | rs10778270 | C/A | 0.228 | 0.222 | 0.825 | 0.631 |
|  |  | 102572584 | rs1609860 | C/A | 0.087 | 0.086 | 0.967 | 0.889 |
|  |  | 102572722 | rs831425 | T/C | 0.192 | 0.172 | 0.092 | 0.116 |
|  |  | 102577550 | rs7315384 | A/C | 0.25 | 0.241 | 0.489 | 0.5 |
|  |  | 102578564 | rs831427 | C/T | 0.173 | 0.185 | 0.415 | 0.35 |
|  |  | 102579218 | rs7958947 | G/A | 0.347 | 0.373 | 0.099 | 0.102 |
|  |  | 102580452 | rs10778272 | G/A | 0.39 | 0.397 | 0.291 | 0.67 |
|  |  | 102582910 | rs10745978 | T/C | 0.4 | 0.423 | 0.311 | 0.138 |
|  |  | 102583920 | rs755598 | A/C | 0.4 | 0.423 | 0.322 | 0.144 |
|  |  | 102585727 | rs7296829 | T/C | 0.4 | 0.426 | 0.259 | 0.104 |
|  |  | 102585961 | rs831429 | A/G | 0.229 | 0.245 | 0.378 | 0.262 |
|  |  | 102587220 | rs1965076 | T/C | 0.468 | 0.463 | 0.568 | 0.802 |
|  |  | 102588826 | rs12424164 | A/G | 0.124 | 0.118 | 0.668 | 0.562 |
|  |  | 102588828 | rs703639 | C/T | 0.176 | 0.19 | 0.598 | 0.26 |
|  |  | 102589976 | rs1863878 | G/A | 0.338 | 0.343 | 0.265 | 0.738 |
|  |  | 102591942 | rs11614418 | C/T | 0.394 | 0.389 | 0.713 | 0.723 |
|  |  | 102592359 | rs703645 | A/G | 0.131 | 0.15 | 0.309 | 0.1 |
|  |  | 102593642 | rs697200 | G/A | 0.386 | 0.382 | 0.721 | 0.77 |
|  |  | 102595653 | rs1593809 | T/C | 0.361 | 0.355 | 0.85 | 0.744 |
|  |  | 102598281 | rs12314920 | A/G | 0.425 | 0.424 | 0.931 | 0.925 |
|  |  | 102605853 | rs11111712 | C/T | 0.152 | 0.168 | 0.389 | 0.194 |
|  |  | 102608711 | rs831423 | G/A | 0.182 | 0.183 | 0.795 | 0.919 |
|  |  | 102608980 | rs11111714 | C/A | 0.067 | 0.071 | 0.72 | 0.638 |
|  |  | 102610170 | rs703649 | G/A | 0.374 | 0.371 | 0.782 | 0.847 |
|  |  | 102611450 | rs703652 | A/G | 0.473 | 0.46 | 0.62 | 0.428 |
|  |  | 102617126 | rs697210 | T/C | 0.274 | 0.289 | 0.656 | 0.337 |
|  |  | 102623284 | rs697211 | G/A | 0.293 | 0.292 | 0.822 | 0.977 |
|  |  | 102626633 | rs6539098 | T/C | 0.246 | 0.238 | 0.613 | 0.608 |
|  |  | 102627186 | rs7310585 | T/C | 0.145 | 0.137 | 0.613 | 0.486 |
|  |  | 102628357 | rs11111722 | C/T | 0.053 | 0.051 | 0.354 | 0.873 |
|  |  | 102636076 | rs6539099 | A/C | 0.157 | 0.154 | 0.839 | 0.785 |
|  |  | 102636357 | rs7975156 | A/G | 0.156 | 0.153 | 0.805 | 0.795 |
|  |  | 102637806 | rs703654 | A/G | 0.203 | 0.192 | 0.548 | 0.412 |
|  |  | 102639543 | rs11111732 | A/G | 0.103 | 0.101 | 0.806 | 0.888 |
|  |  | 102641592 | rs11111736 | G/A | 0.037 | 0.036 | 0.343 | 0.897 |
|  |  | 102642509 | rs10861081 | C/A | 0.102 | 0.099 | 0.433 | 0.755 |
|  |  | 102642717 | rs2270485 | A/G | 0.046 | 0.046 | 0.591 | 0.96 |
|  |  | 102647673 | rs10861083 | A/C | 0.159 | 0.156 | 0.931 | 0.809 |
|  |  | 102648191 | rs4981042 | G/A | 0.323 | 0.331 | 0.402 | 0.628 |
|  |  | 102650798 | rs17034439 | C/A | 0.12 | 0.128 | 0.418 | 0.438 |
|  |  | 102652796 | rs11111738 | T/C | 0.169 | 0.175 | 0.323 | 0.66 |
|  |  | 102653653 | rs1946821 | A/C | 0.458 | 0.475 | 0.056 | 0.327 |
|  |  | 102653932 | rs4981030 | G/A | 0.387 | 0.4 | 0.196 | 0.423 |
|  |  | 102654640 | rs10778284 | G/A | 0.19 | 0.197 | 0.52 | 0.586 |
|  |  | 102654977 | rs17505525 | A/G | 0.112 | 0.115 | 0.522 | 0.82 |
|  |  | 102656495 | rs1370785 | G/A | 0.156 | 0.16 | 0.951 | 0.72 |
|  |  | 102659213 | rs3844213 | A/G | 0.26 | 0.266 | 0.452 | 0.656 |
|  |  | 102660014 | rs7301863 | C/T | 0.402 | 0.383 | 0.621 | 0.234 |
|  |  | 102661440 | rs12422579 | G/A | 0.061 | 0.058 | 0.05 | 0.662 |
|  |  | 102663048 | rs2292687 | C/T | 0.241 | 0.207 | 4.62 10^-3^ | 0.014 |
|  |  | 102663164 | rs7306642 | C/A | 0.068 | 0.074 | 0.586 | 0.483 |
|  |  | 102664647 | rs2056128 | G/A | 0.42 | 0.449 | 0.131 | 0.07 |
|  |  | 102667212 | rs7138637 | G/A | 0.07 | 0.078 | 0.954 | 0.346 |
|  |  | 102668965 | rs17505851 | G/A | 0.234 | 0.271 | 8.69 10^-3^ | 0.011 |
|  |  | 102670473 | rs12423929 | G/A | 0.057 | 0.063 | 0.78 | 0.469 |
|  |  | 102670503 | rs12426526 | A/G | 0.061 | 0.066 | 0.844 | 0.545 |
|  |  | 102671712 | rs11111747 | A/G | 0.24 | 0.274 | 0.022 | 0.017 |
|  |  | 102673152 | rs11111748 | A/G | 0.05 | 0.056 | 0.715 | 0.362 |
|  |  | 102673650 | rs7313163 | T/G | 0.347 | 0.355 | 0.179 | 0.583 |
|  |  | 102674004 | rs4981022 | A/G | 0.295 | 0.332 | 0.036 | 0.016 |
|  |  | 102674129 | rs4981021 | C/T | 0.302 | 0.25 | 3.17 10^-4^ | 5.25 10^-4^ |
|  |  | 102675690 | rs11834389 | T/C | 0.095 | 0.094 | 0.714 | 0.926 |
|  |  | 102681632 | rs3751196 | G/A | 0.075 | 0.075 | 0.319 | 0.982 |
|  | WDR66^(D2)^ | 120849966 | rs7961894 | C/T | 0.101 | 0.118 | 0.128 | 0.099 |
|  |  | 120862589 | rs895958 | A/G | 0.121 | 0.13 | 0.728 | 0.416 |
|  |  | 120866612 | rs1720037 | T/G | 0.465 | 0.469 | 0.812 | 0.791 |
|  |  | 120872697 | rs1375653 | G/A | 0.225 | 0.232 | 0.219 | 0.604 |
|  |  | 120876421 | rs11043265 | C/T | 0.059 | 0.044 | 0.695 | 0.052 |
|  |  | 120878459 | rs7139277 | A/G | 0.053 | 0.042 | 0.789 | 0.123 |
|  |  | 120885884 | rs1169072 | G/A | 0.437 | 0.439 | 0.977 | 0.911 |
|  |  | 120890295 | rs1169081 | G/T | 0.308 | 0.301 | 0.623 | 0.641 |
|  |  | 120910600 | rs830123 | G/A | 0.152 | 0.152 | 0.979 | 0.966 |
|  |  | 120913867 | rs1169076 | A/G | 0.222 | 0.232 | 0.137 | 0.475 |
|  | STX2^(D1)^ | 129818504 | rs12318582 | C/T | 0.15 | 0.155 | 0.377 | 0.616 |
|  |  | 129818894 | rs4759515 | C/T | 0.312 | 0.321 | 0.624 | 0.55 |
|  |  | 129834650 | rs2632601 | C/T | 0.392 | 0.401 | 0.944 | 0.605 |
|  |  | 129837246 | rs2632667 | A/G | 0.476 | 0.475 | 0.98 | 0.956 |
|  |  | 129843724 | rs10219721 | C/A | 0.099 | 0.089 | 0.628 | 0.312 |
|  |  | 129844975 | rs10773819 | A/G | 0.477 | 0.445 | 0.183 | 0.044 |
|  |  | 129846370 | rs1554807 | C/T | 0.377 | 0.356 | 0.395 | 0.202 |
|  |  | 129853124 | rs12369479 | T/C | 0.047 | 0.056 | 0.911 | 0.202 |
|  |  | 129866048 | rs1106369 | A/C | 0.477 | 0.445 | 0.212 | 0.051 |
|  |  | 129880167 | rs4334059 | C/T | 0.37 | 0.354 | 0.561 | 0.324 |
|  |  | 129880774 | rs4644680 | G/A | 0.217 | 0.195 | 0.154 | 0.111 |
|  |  | 129887167 | rs7311067 | C/T | 0.241 | 0.241 | 0.736 | 1 |
|  |  | 129890817 | rs7962097 | G/A | 0.197 | 0.175 | 0.299 | 0.097 |
|  |  | 129896126 | rs4420312 | C/T | 0.231 | 0.226 | 0.969 | 0.72 |
|  |  | 129898017 | rs10848218 | C/T | 0.419 | 0.441 | 0.579 | 0.183 |
| 13 | CPB2  ^(B)^ | 45527945 | rs1926447 | G/A | 0.308 | 0.325 | 0.047 | 0.273 |
|  |  | 45528642 | rs7989892 | T/C | 0.269 | 0.289 | 0.025 | 0.188 |
|  |  | 45534236 | rs2897027 | G/A | 0.269 | 0.289 | 0.025 | 0.177 |
|  |  | 45539686 | rs9316180 | A/G | 0.326 | 0.31 | 0.158 | 0.298 |
|  |  | 45541562 | rs7993537 | A/G | 0.326 | 0.31 | 0.164 | 0.291 |
|  |  | 45543741 | rs9316181 | G/A | 0.326 | 0.31 | 0.154 | 0.282 |
|  |  | 45553450 | rs723391 | T/C | 0.357 | 0.336 | 0.167 | 0.18 |
|  |  | 45553502 | rs1409433 | C/T | 0.27 | 0.292 | 0.027 | 0.146 |
|  |  | 45554413 | rs3818477 | T/G | 0.374 | 0.374 | 0.537 | 0.977 |
|  |  | 45566303 | rs1409432 | A/G | 0.27 | 0.285 | 0.054 | 0.316 |
|  |  | 45571318 | rs2181617 | G/A | 0.491 | 0.459 | 0.08 | 0.052 |
|  |  | 45572910 | rs1952187 | G/A | 0.344 | 0.324 | 0.141 | 0.215 |
|  |  | 45573715 | rs17600984 | C/A | 0.244 | 0.26 | 0.943 | 0.255 |
|  |  | 45574196 | rs17067700 | G/A | 0.409 | 0.413 | 0.125 | 0.815 |
|  | F7^(A)^ | 112800832 | rs10665 | A/G | 0.137 | 0.148 | 0.034 | 0.348 |
|  |  | 112801165 | rs2181540 | T/C | 0.143 | 0.149 | 0.061 | 0.65 |
|  |  | 112804541 | rs555212 | G/A | 0.201 | 0.222 | 0.521 | 0.127 |
|  |  | 112811927 | rs1475931 | G/T | 0.256 | 0.221 | 0.087 | 0.013 |
|  |  | 112818877 | rs488703 | G/A | 0.122 | 0.132 | 0.035 | 0.401 |
|  | F10^(A)^ | 112825510 | rs3211719 | A/G | 0.228 | 0.215 | 0.149 | 0.328 |
|  |  | 112828042 | rs556694 | T/C | 0.106 | 0.114 | 0.044 | 0.423 |
|  |  | 112835822 | rs513101 | A/G | 0.145 | 0.153 | 0.472 | 0.5 |
|  |  | 112841850 | rs3211770 | G/A | 0.128 | 0.132 | 0.21 | 0.732 |
|  |  | 112849738 | rs5960 | T/C | 0.123 | 0.128 | 0.375 | 0.645 |
| 14 | TC2N^(D1)^ | 91311785 | rs4243690 | G/A | 0.026 | 0.034 | 0.106 | 0.152 |
|  |  | 91360497 | rs10498632 | A/G | 0.357 | 0.337 | 0.188 | 0.186 |
|  |  | 91363271 | rs2402074 | G/A | 0.475 | 0.437 | 0.053 | 0.021 |
|  |  | 91378982 | rs1884841 | G/A | 0.474 | 0.438 | 0.072 | 0.026 |
|  |  | 91384529 | rs2402075 | A/C | 0.03 | 0.037 | 0.229 | 0.25 |
| 16 | MBTPS1 ^(D5)^ | 82649967 | rs2280025 | C/T | 0.273 | 0.278 | 0.397 | 0.725 |
|  |  | 82650563 | rs2280024 | A/G | 0.474 | 0.428 | 0.018 | 4.37 10^-3^ |
|  |  | 82651198 | rs4782888 | C/A | 0.046 | 0.06 | 0.193 | 0.053 |
|  |  | 82652366 | rs999783 | C/T | 0.274 | 0.28 | 0.394 | 0.713 |
|  |  | 82654078 | rs883635 | A/G | 0.238 | 0.279 | 0.035 | 4.07 10^-3^ |
|  |  | 82654806 | rs2280022 | T/G | 0.28 | 0.283 | 0.507 | 0.812 |
|  |  | 82656116 | rs10445073 | A/G | 0.251 | 0.278 | 0.254 | 0.073 |
|  |  | 82659376 | rs3817151 | C/T | 0.448 | 0.489 | 0.043 | 0.011 |
|  |  | 82661979 | rs1980011 | T/C | 0.334 | 0.304 | 0.036 | 0.05 |
|  |  | 82667498 | rs2175248 | A/G | 0.132 | 0.169 | 0.012 | 2.09 10^-3^ |
|  |  | 82672780 | rs3826139 | T/C | 0.298 | 0.299 | 0.689 | 0.941 |
|  |  | 82678157 | rs2875857 | A/C | 0.417 | 0.408 | 0.706 | 0.558 |
|  |  | 82682771 | rs8050801 | A/G | 0.14 | 0.121 | 0.331 | 0.09 |
|  |  | 82683859 | rs11149625 | C/A | 0.29 | 0.294 | 0.649 | 0.827 |
|  |  | 82689206 | rs11647035 | T/C | 0.055 | 0.045 | 0.17 | 0.152 |
|  |  | 82690034 | rs4782584 | G/A | 0.354 | 0.354 | 0.353 | 0.976 |
|  |  | 82690941 | rs17726874 | A/G | 0.293 | 0.281 | 0.193 | 0.413 |
|  |  | 82692994 | rs907045 | G/A | 0.284 | 0.306 | 0.412 | 0.15 |
|  |  | 82694123 | rs3785028 | C/T | 0.27 | 0.26 | 0.219 | 0.522 |
| 17 | SERPINF2  ^(B)^ | 1595044 | rs2070862 | C/T | 0.207 | 0.224 | 0.633 | 0.213 |
|  |  | 1598530 | rs2277695 | G/A | 0.303 | 0.255 | 2.18 10^-3^ | 1.27 10^-3^ |
|  |  | 1603823 | rs8074026 | C/T | 0.295 | 0.243 | 6.87 10^-4^ | 3.85 10^-4^ |
|  |  | 1607361 | rs17761310 | C/T | 0.071 | 0.095 | 0.089 | 0.01 |
|  |  | 1607551 | rs8075977 | T/C | 0.402 | 0.405 | 0.357 | 0.838 |
|  | SERPINF1  ^(B)^ | 1620026 | rs1136287 | T/C | 0.327 | 0.328 | 0.388 | 0.945 |
|  | TAOK1^(D2)^ | 24777744 | rs6505129 | A/G | 0.496 | 0.478 | 0.061 | 0.282 |
|  |  | 24777972 | rs883828 | C/A | 0.164 | 0.175 | 0.152 | 0.375 |
|  |  | 24793724 | rs9900280 | A/G | 0.498 | 0.478 | 0.057 | 0.243 |
|  |  | 24799793 | rs7217677 | C/A | 0.164 | 0.175 | 0.149 | 0.369 |
|  |  | 24802960 | rs4795510 | A/G | 0.496 | 0.477 | 0.05 | 0.251 |
|  |  | 24856121 | rs490120 | A/G | 0.27 | 0.29 | 0.715 | 0.167 |
|  |  | 24859695 | rs1529420 | T/C | 0.101 | 0.121 | 0.088 | 0.048 |
|  |  | 24865310 | rs9898519 | A/G | 0.234 | 0.225 | 0.874 | 0.528 |
|  |  | 24885455 | rs3744624 | T/C | 0.203 | 0.201 | 0.977 | 0.876 |
|  |  | 24896273 | rs602056 | C/T | 0.497 | 0.477 | 0.039 | 0.216 |
|  |  | 24899339 | rs11868320 | G/A | 0.224 | 0.187 | 6.49 10^-3^ | 5.99 10^-3^ |
|  |  | 24900687 | rs2559619 | A/G | 0.272 | 0.291 | 0.743 | 0.189 |
| 19 | CLEC4M^(D1)^ | 7733137 | rs7252764 | C/T | 0.042 | 0.043 | 0.248 | 0.87 |
|  |  | 7733830 | rs571497 | G/A | 0.182 | 0.164 | 0.829 | 0.155 |
|  |  | 7733955 | rs2287887 | A/C | 0.29 | 0.285 | 0.32 | 0.716 |
|  |  | 7737166 | rs868875 | A/G | 0.329 | 0.343 | 0.624 | 0.354 |
|  |  | 7737953 | rs560634 | G/T | 0.154 | 0.135 | 0.512 | 0.106 |
|  |  | 7739690 | rs3745376 | G/T | 0.149 | 0.164 | 0.452 | 0.238 |
|  |  | 7740274 | rs15282 | C/T | 0.344 | 0.341 | 0.434 | 0.862 |
|  |  | 7743306 | rs608773 | G/A | 0.499 | 0.499 | 0.138 | 0.982 |
|  |  | 7743743 | rs621025 | G/T | 0.353 | 0.366 | 0.153 | 0.414 |
|  |  | 7744484 | rs807969 | G/T | 0.168 | 0.172 | 0.353 | 0.759 |
|  |  | 7746310 | rs4804814 | C/T | 0.238 | 0.221 | 0.359 | 0.23 |
|  | GP6 ^(C)^ | 60216625 | rs1654410 | T/C | 0.484 | 0.488 | 0.169 | 0.807 |
|  |  | 60216717 | rs10416380 | G/A | 0.141 | 0.155 | 0.087 | 0.253 |
|  |  | 60227224 | rs11084381 | C/T | 0.15 | 0.161 | 0.078 | 0.324 |
|  |  | 60227784 | rs11669150 | T/C | 0.246 | 0.251 | 0.333 | 0.762 |
|  |  | 60228407 | rs1613662 | A/G | 0.131 | 0.149 | 0.018 | 0.115 |
|  |  | 60231360 | rs1671196 | T/C | 0.149 | 0.163 | 0.063 | 0.269 |
| 20 | THBD ^(A)^ | 22973295 | rs2007363 | G/T | 0.197 | 0.196 | 0.888 | 0.923 |
|  |  | 22975413 | rs3176123 | T/G | 0.147 | 0.174 | 0.22 | 0.023 |
|  |  | 22982274 | rs6048519 | T/G | 0.36 | 0.372 | 0.376 | 0.453 |
|  |  | 22984049 | rs2424505 | G/A | 0.077 | 0.089 | 0.336 | 0.176 |
|  |  | 22989366 | rs1318041 | A/G | 0.223 | 0.245 | 0.468 | 0.121 |
|  | EDEM2 ^(D3)^ | 33157518 | rs6088713 | C/T | 0.251 | 0.281 | 0.093 | 0.041 |
|  |  | 33158241 | rs6060239 | T/C | 0.152 | 0.129 | 0.024 | 0.047 |
|  |  | 33167268 | rs3746429 | C/T | 0.209 | 0.178 | 0.017 | 0.019 |
|  |  | 33169672 | rs6088721 | C/A | 0.366 | 0.369 | 1 | 0.837 |
|  |  | 33169848 | rs6088722 | C/T | 0.252 | 0.28 | 0.095 | 0.051 |
|  |  | 33177300 | rs6088727 | A/G | 0.368 | 0.371 | 0.914 | 0.826 |
|  |  | 33182367 | rs1535466 | A/G | 0.209 | 0.243 | 0.08 | 0.013 |
|  |  | 33198154 | rs1415771 | G/A | 0.46 | 0.449 | 0.424 | 0.497 |
|  |  | 33200475 | rs6060270 | G/T | 0.275 | 0.23 | 2.01 10^-3^ | 1.66 10^-3^ |
|  |  | 33209337 | rs6088735 | C/T | 0.282 | 0.232 | 7.36 10^-4^ | 6.10 10^-4^ |
|  | PROCR^(A,D3)^ | 33216923 | rs6060278 | T/C | 0.281 | 0.232 | 6.26 10^-4^ | 7.31 10^-4^ |
|  |  | 33218066 | rs8119351 | G/A | 0.113 | 0.09 | 0.019 | 0.025 |
|  |  | 33228215 | rs867186 | A/G | 0.116 | 0.097 | 0.058 | 0.072 |
|  |  | 33241644 | rs6058202 | G/A | 0.423 | 0.456 | 0.025 | 0.05 |
|  |  | 33252704 | rs6142324 | C/T | 0.427 | 0.458 | 0.023 | 0.059 |
|  | MMP24 ^(D3)^ | 33261149 | rs6120870 | A/G | 0.129 | 0.109 | 0.101 | 0.065 |
|  |  | 33282831 | rs2425019 | G/A | 0.485 | 0.468 | 7.49 10^-3^ | 5.13 10^-3^ |
|  |  | 33288794 | rs12479765 | G/A | 0.235 | 0.198 | 5.79 10^-3^ | 5.52 10^-3^ |
|  |  | 33294603 | rs2425022 | T/C | 0.15 | 0.165 | 0.163 | 0.214 |
|  |  | 33299189 | rs6088776 | T/C | 0.183 | 0.156 | 0.028 | 0.031 |
|  |  | 33303355 | rs2247828 | A/G | 0.134 | 0.147 | 0.204 | 0.23 |
|  |  | 33308354 | rs2425024 | A/C | 0.38 | 0.356 | 0.143 | 0.128 |
|  |  | 33312595 | rs1555322 | G/A | 0.128 | 0.142 | 0.179 | 0.202 |
|  |  | 33320958 | rs2275274 | C/T | 0.105 | 0.087 | 0.088 | 0.06 |
