# Supplementary material for: Genetics of Venous Thrombosis: Insights from a New Genome Wide Association Study
Source: PLoS One. 2011 Sep 27;6(9):e25581. doi: 10.1371/journal.pone.0025581 (PMC3181335; doi:10.1371/journal.pone.0025581)
Supplement: Table S2 — Haplotype structure derived from VT-associated F11 SNPs in the HapMap database. Haplotype frequencies were estimated using the Haploview software from the HapMap 3 (release 2) data. SNPs identified in the LETS study (Li Y et al. J Thromb Haemost 2009;7:1802–1808) are shown in bold, others were those identified in the current MARTHA project. (DOC) [file pone.0025581.s002.doc]

**Table S2.** Haplotype structure derived from VT-associated *F11* SNPs in the HapMap database.

| Polymorphisms | | | | | | Haplotype Frequencies |
| --- | --- | --- | --- | --- | --- | --- |
| rs925451 | **rs2036914** | **rs2289252** | rs10029715 | rs1008728 | rs13133050 | HapMap data |
| A | **C** | **T** | T | T | C | 0.308 |
| A | **C** | **T** | T | C | A | 0.024 |
| A | **C** | **T** | C | C | C | 0.011 |
| G | **C** | **T** | T | T | C | 0.141 |
| G | **C** | **C** | T | T | C | 0.112 |
| G | **T** | **T** | T | T | C | 0.030 |
| G | **T** | **C** | T | C | A | 0.138 |
| G | **C** | **C** | T | C | A | 0.022 |
| G | **T** | **C** | C | C | C | 0.048 |
| G | **T** | **C** | C | C | A | 0.102 |
