# Supplementary material for: Genetics of Venous Thrombosis: Insights from a New Genome Wide Association Study
Source: PLoS One. 2011 Sep 27;6(9):e25581. doi: 10.1371/journal.pone.0025581 (PMC3181335; doi:10.1371/journal.pone.0025581)
Supplement: Table S3 — Stage II - Genome-wide significant (p<2.01 10−8) SNP imputed associations with VT in the combined discovery and in silico GWASes of 1,961 cases and 2,338 controls. (1) Common/rare alleles. (2) Minor allele frequency. (3) Odds Ratio associated with the minor allele estimated from the Mach2dat imputation software, after adjusting for principal components. (4) Combined p-values computed using the inverse-variance model as implemented in METAL software. All shown imputed SNPs satisfied the imputation quality criteria (r2.hat>0.3). (DOC) [file pone.0025581.s003.doc]

**Table S3.** Stage II - Genome-wide significant (p <2.01 10^-8^) SNP imputed associations with VT in the combined discovery and *in silico* GWASes of 1,961 cases and 2,338 controls.

| CHR | Position (Build 35) | SNPID | Allele^(1)^ | Discovery GWAS | | | In silico GWAS | | | Meta-Analysis | |
| --- | --- | --- | --- | --- | --- | --- | --- | --- | --- | --- | --- |
|  |  |  |  | MAF^(2)^ | OR^(3)^ | P | MAF | OR | P | OR | P^(4)^ |
| 1 | 165866785 | rs16861990 | A/C | 0.101 | 2.49 | 1.363 x 10^-16^ | 0.071 | 2.13 | 3.156 x 10^-6^ | 2.36 | 4.896 x 10^-20^ |
| 1 | 166028522 | rs1208327 | T/C | 0.087 | 3.12 | 4.641 x 10^-18^ | 0.060 | 2.66 | 1.99 x 10^-7^ | 2.95 | 1.452 x 10^-22^ |
| 1 | 166083202 | rs7538157 | A/C | 0.053 | 5.30 | 1.04 x 10^-19^ | 0.030 | 5.27 | 2.765 x 10^-9^ | 5.29 | 9.029 x 10^-25^ |
| 1 | 166155756 | rs1208135 | G/A | 0.099 | 2.53 | 9.926 x 10^-17^ | 0.072 | 2.12 | 1.815 x 10^-6^ | 2.37 | 2.123 x 10^-20^ |
| 1 | 166160602 | rs1208134 | T/C | 0.099 | 2.53 | 9.831 x 10^-17^ | 0.072 | 2.12 | 1.809 x 10^-6^ | 2.37 | 2.123 x 10^-20^ |
| 1 | 166192384 | rs6696217 | G/A | 0.139 | 1.92 | 7.481 x 10^-11^ | 0.113 | 1.70 | 0.0001982 | 1.84 | 1.377 x 10^-13^ |
| 1 | 166199312 | rs1894692 | A/G | 0.016 | 59.5 | 4.834 x 10^-15^ | 0.008 | 16.9 | 0.0002573 | 36.9 | 1.79 x 10^-13^ |
| 1 | 166207710 | rs10737547 | G/A | 0.109 | 2.64 | 8.388 x 10^-18^ | 0.085 | 2.25 | 1.654 x 10^-7^ | 2.49 | 1.487 x 10^-22^ |
| 1 | 166209232 | rs6687813 | C/A | 0.109 | 2.64 | 8.318 x 10^-18^ | 0.085 | 2.25 | 1.634 x 10^-7^ | 2.49 | 8.606 x 10^-23^ |
| 1 | 166211632 | rs970740 | T/C | 0.116 | 2.61 | 3.743 x 10^-20^ | 0.090 | 2.30 | 1.013 x 10^-8^ | 2.49 | 4.474 x 10^-26^ |
| 1 | 166212779 | rs6427194 | A/T | 0.116 | 2.61 | 3.67 x 10^-20^ | 0.090 | 2.30 | 1.016 x 10^-8^ | 2.49 | 5.059 x 10^-26^ |
| 1 | 166212834 | rs6427195 | T/A | 0.116 | 2.61 | 3.644 x 10^-20^ | 0.090 | 2.30 | 1.02 x 10^-8^ | 2.49 | 5.059 x 10^-26^ |
| 1 | 166212881 | rs6427196 | G/C | 0.116 | 2.61 | 3.639 x 10^-20^ | 0.090 | 2.29 | 1.027 x 10^-8^ | 2.49 | 5.292 x 10^-26^ |
| 1 | 166222050 | rs2420370 | C/G | 0.115 | 2.62 | 2.479 x 10^-20^ | 0.090 | 2.28 | 1.101 x 10^-8^ | 2.49 | 4.15 x 10^-26^ |
| 1 | 166223213 | rs2420371 | A/G | 0.115 | 2.62 | 2.23 x 10^-20^ | 0.090 | 2.28 | 1.128 x 10^-8^ | 2.49 | 4.02 x 10^-26^ |
| 1 | 166229714 | rs2420372 | G/A | 0.116 | 2.62 | 2.274 x 10^-20^ | 0.090 | 2.30 | 1.031 x 10^-8^ | 2.50 | 3.386 x 10^-26^ |
| 1 | 166230492 | rs6009 | C/T | 0.116 | 2.63 | 2.28 x 10^-20^ | 0.090 | 2.30 | 1.023 x 10^-8^ | 2.50 | 3.964 x 10^-26^ |
| 1 | 166232248 | rs6427197 | A/C | 0.116 | 2.62 | 2.451 x 10^-20^ | 0.090 | 2.31 | 9.647 x 10^-9^ | 2.50 | 3.748 x 10^-26^ |
| 1 | 166245664 | rs1018827 | G/A | 0.116 | 2.62 | 2.867 x 10^-20^ | 0.089 | 2.35 | 5.825 x 10^-9^ | 2.52 | 2.41 x 10^-26^ |
| 1 | 166250707 | rs6025 | C/T | 0.017 | 70.6 | 5.639 x 10^-17^ | 0.008 | 20.3 | 2.932 x 10^-5^ | 42.1 | 3.726 x 10^-15^ |
| 4 | 155858793 | rs6825454 | T/C | 0.268 | 1.50 | 1.899 x 10^-9^ | 0.264 | 1.42 | 0.0003373 | 1.47 | 4.32 x 10^-12^ |
| 4 | 155865195 | rs6050 | T/C | 0.267 | 1.51 | 1.22 x 10^-9^ | 0.262 | 1.45 | 0.0001821 | 1.49 | 1.412 x 10^-12^ |
| 4 | 155872484 | rs13109457 | G/A | 0.267 | 1.51 | 1.116 x 10^-9^ | 0.262 | 1.45 | 0.0001676 | 1.49 | 1.299 x 10^-12^ |
| 4 | 155878535 | rs7659024 | G/A | 0.251 | 1.56 | 2.487 x 10^-10^ | 0.243 | 1.47 | 0.0001356 | 1.53 | 1.925 x 10^-13^ |
| 4 | 155882881 | rs2066865 | G/A | 0.250 | 1.55 | 2.611 x 10^-10^ | 0.243 | 1.47 | 0.0001355 | 1.53 | 2.286 x 10^-13^ |
| 4 | 155883300 | rs2066864 | G/A | 0.251 | 1.55 | 2.774 x 10^-10^ | 0.243 | 1.47 | 0.0001345 | 1.53 | 2.286 x 10^-13^ |
| 4 | 155885041 | rs2066861 | C/T | 0.251 | 1.55 | 3.142 x 10^-10^ | 0.243 | 1.47 | 0.0001335 | 1.52 | 3.014 x 10^-13^ |
| 4 | 155894926 | rs12644950 | G/A | 0.252 | 1.55 | 3.529 x 10^-10^ | 0.243 | 1.47 | 0.0001318 | 1.53 | 2.773 x 10^-13^ |
| 4 | 155896075 | rs13130318 | T/G | 0.243 | 1.54 | 4.023 x 10^-9^ | 0.233 | 1.50 | 0.0002153 | 1.53 | 4.524 x 10^-12^ |
| 4 | 155899853 | rs7681423 | C/T | 0.252 | 1.55 | 3.512 x 10^-10^ | 0.243 | 1.49 | 0.0001303 | 1.53 | 4.047 x 10^-13^ |
| 4 | 155902677 | rs7654093 | A/T | 0.252 | 1.58 | 3.654 x 10^-10^ | 0.243 | 1.49 | 0.0001303 | 1.54 | 3.672 x 10^-13^ |
| 4 | 187497468 | rs3736455 | G/T | 0.342 | 0.75 | 1.355 x 10^-5^ | 0.340 | 0.69 | 9.666 x 10^-5^ | 0.73 | 9.194 x 10^-9^ |
| 4 | 187560534 | rs3756008 | A/T | 0.436 | 1.36 | 3.414 x 10^-7^ | 0.394 | 1.47 | 2.613 x 10^-5^ | 1.40 | 6.462 x 10^-11^ |
| 4 | 187561260 | rs3756009 | A/G | 0.431 | 1.34 | 1.132 x 10^-6^ | 0.385 | 1.44 | 6.58 x 10^-5^ | 1.37 | 4.929 x 10^-10^ |
| 4 | 187562718 | rs925451 | G/A | 0.431 | 1.34 | 1.185 x 10^-6^ | 0.385 | 1.44 | 6.388 x 10^-5^ | 1.37 | 5.377 x 10^-10^ |
| 4 | 187563243 | rs4253399 | T/G | 0.438 | 1.34 | 9.217 x 10^-7^ | 0.395 | 1.45 | 3.112 x 10^-5^ | 1.38 | 2.037 x 10^-10^ |
| 4 | 187566936 | rs4241824 | A/G | 0.469 | 0.77 | 3.537 x 10^-5^ | 0.485 | 0.64 | 1.43 x 10^-6^ | 0.73 | 1.403 x 10^-9^ |
| 4 | 187567630 | rs2036914 | C/T | 0.434 | 0.76 | 1.726 x 10^-5^ | 0.461 | 0.65 | 3.442 x 10^-6^ | 0.72 | 1.04 x 10^-9^ |
| 4 | 187570700 | rs1593 | A/T | 0.096 | 0.53 | 1.471 x 10^-8^ | 0.097 | 0.55 | 0.0007667 | 0.54 | 1.009 x 10^-10^ |
| 4 | 187574154 | rs4253417 | T/C | 0.437 | 1.37 | 5.765 x 10^-7^ | 0.402 | 1.43 | 0.00021 | 1.39 | 6.965 x 10^-10^ |
| 4 | 187593872 | rs11132387 | G/A | 0.476 | 1.34 | 1.374 x 10^-5^ | 0.468 | 1.50 | 8.494 x 10^-5^ | 1.39 | 9.194 x 10^-9^ |
| 4 | 187595896 | rs907439 | C/T | 0.144 | 0.62 | 1.081 x 10^-7^ | 0.147 | 0.67 | 0.003242 | 0.64 | 1.951 x 10^-9^ |
| 4 | 187597749 | rs13351601 | T/C | 0.143 | 0.62 | 9.254 x 10^-8^ | 0.147 | 0.67 | 0.003196 | 0.63 | 1.556 x 10^-9^ |
| 4 | 187598732 | rs4572916 | A/C | 0.143 | 0.62 | 9.007 x 10^-8^ | 0.147 | 0.67 | 0.003189 | 0.63 | 1.519 x 10^-9^ |
| 4 | 187600453 | rs10025152 | G/A | 0.143 | 0.62 | 8.305 x 10^-8^ | 0.147 | 0.66 | 0.0031 | 0.63 | 1.319 x 10^-9^ |
| 4 | 187601822 | rs1008728 | T/C | 0.368 | 0.74 | 2.434 x 10^-6^ | 0.363 | 0.67 | 5.39 x 10^-5^ | 0.72 | 1.274 x 10^-9^ |
| 4 | 187602600 | rs12500826 | C/T | 0.368 | 0.74 | 2.785 x 10^-6^ | 0.363 | 0.67 | 5.458 x 10^-5^ | 0.72 | 1.386 x 10^-9^ |
| 9 | 133161857 | rs8176732 | A/G | 0.211 | 0.70 | 3.147 x 10^-6^ | 0.224 | 0.68 | 0.0007392 | 0.70 | 1.375 x 10^-8^ |
| 9 | 133162115 | rs8176728 | G/C | 0.211 | 0.70 | 3.141 x 10^-6^ | 0.224 | 0.68 | 0.000747 | 0.70 | 1.05 x 10^-8^ |
| 9 | 133162261 | rs2073825 | A/T | 0.211 | 0.70 | 3.177 x 10^-6^ | 0.224 | 0.68 | 0.0007489 | 0.70 | 1.154 x 10^-8^ |
| 9 | 133162588 | rs8176717 | G/T | 0.211 | 0.70 | 3.181 x 10^-6^ | 0.224 | 0.68 | 0.0007296 | 0.70 | 1.2 x 10^-8^ |
| 9 | 133162732 | rs8176714 | G/A | 0.211 | 0.70 | 3.188 x 10^-6^ | 0.224 | 0.68 | 0.0007203 | 0.70 | 1.167 x 10^-8^ |
| 9 | 133163253 | rs641959 | A/C | 0.232 | 0.66 | 1.227 x 10^-8^ | 0.250 | 0.65 | 8.3 x 10^-5^ | 0.66 | 6.396 x 10^-12^ |
| 9 | 133163268 | rs641943 | A/G | 0.232 | 0.66 | 1.233 x 10^-8^ | 0.250 | 0.65 | 8.516 x 10^-5^ | 0.66 | 6.938 x 10^-12^ |
| 9 | 133163297 | rs514708 | C/T | 0.232 | 0.66 | 1.24 x 10^-8^ | 0.250 | 0.65 | 8.657 x 10^-5^ | 0.66 | 7.184 x 10^-12^ |
| 9 | 133163588 | rs517414 | G/A | 0.211 | 0.70 | 3.223 x 10^-6^ | 0.224 | 0.68 | 0.0007605 | 0.70 | 1.318 x 10^-8^ |
| 9 | 133164026 | rs638756 | A/C | 0.218 | 0.69 | 7.483 x 10^-7^ | 0.233 | 0.65 | 0.0002272 | 0.68 | 1.158 x 10^-9^ |
| 9 | 133164548 | rs626035 | T/G | 0.211 | 0.70 | 3.254 x 10^-6^ | 0.224 | 0.68 | 0.0007984 | 0.70 | 1.394 x 10^-8^ |
| 9 | 133164601 | rs547643 | C/T | 0.211 | 0.70 | 3.257 x 10^-6^ | 0.224 | 0.68 | 0.0008148 | 0.70 | 1.394 x 10^-8^ |
| 9 | 133164650 | rs625593 | G/A | 0.211 | 0.70 | 3.272 x 10^-6^ | 0.224 | 0.68 | 0.0008314 | 0.70 | 1.434 x 10^-8^ |
| 9 | 133164749 | rs549331 | C/G | 0.211 | 0.70 | 3.279 x 10^-6^ | 0.224 | 0.69 | 0.0008798 | 0.70 | 1.516 x 10^-8^ |
| 9 | 133164792 | rs549446 | C/T | 0.211 | 0.70 | 3.296 x 10^-6^ | 0.224 | 0.69 | 0.0008926 | 0.70 | 1.516 x 10^-8^ |
| 9 | 133164919 | rs624601 | G/A | 0.211 | 0.70 | 3.306 x 10^-6^ | 0.224 | 0.69 | 0.0009121 | 0.70 | 1.558 x 10^-8^ |
| 9 | 133165032 | rs613423 | G/A | 0.211 | 0.71 | 3.337 x 10^-6^ | 0.224 | 0.69 | 0.000952 | 0.70 | 1.712 x 10^-8^ |
| 9 | 133165213 | rs574347 | T/C | 0.211 | 0.71 | 3.352 x 10^-6^ | 0.224 | 0.69 | 0.0009876 | 0.70 | 1.809 x 10^-8^ |
| 9 | 133165750 | rs579483 | T/A | 0.210 | 0.70 | 2.997 x 10^-6^ | 0.224 | 0.69 | 0.0009439 | 0.70 | 1.5 x 10^-8^ |
| 9 | 133165796 | rs579622 | G/A | 0.210 | 0.70 | 2.685 x 10^-6^ | 0.224 | 0.69 | 0.0009165 | 0.70 | 1.278 x 10^-8^ |
| 9 | 133166324 | rs688976 | C/A | 0.210 | 0.70 | 2.17 x 10^-6^ | 0.224 | 0.68 | 0.0008851 | 0.70 | 9.252 x 10^-9^ |
| 9 | 133166619 | rs687621 | A/G | 0.433 | 1.84 | 6.587 x 10^-22^ | 0.395 | 2.06 | 1.703 x 10^-15^ | 1.91 | 3.272 x 10^-34^ |
| 9 | 133166660 | rs687289 | G/A | 0.433 | 1.84 | 6.184 x 10^-22^ | 0.395 | 2.06 | 1.646 x 10^-15^ | 1.91 | 2.803 x 10^-34^ |
| 9 | 133166687 | rs2073827 | G/C | 0.356 | 0.70 | 3.221 x 10^-8^ | 0.381 | 0.59 | 6.479 x 10^-8^ | 0.67 | 7.381 x 10^-14^ |
| 9 | 133167679 | rs672316 | T/G | 0.231 | 0.65 | 5.631 x 10^-9^ | 0.249 | 0.65 | 0.0001002 | 0.65 | 3.398 x 10^-12^ |
| 9 | 133168819 | rs657152 | C/A | 0.454 | 1.70 | 2.003 x 10^-17^ | 0.420 | 1.96 | 1.095 x 10^-13^ | 1.78 | 5.462 x 10^-28^ |
| 9 | 133168851 | rs8176682 | C/T | 0.335 | 0.74 | 4.374 x 10^-6^ | 0.355 | 0.62 | 9.288 x 10^-7^ | 0.70 | 1.015 x 10^-10^ |
| 9 | 133169171 | rs474279 | C/T | 0.210 | 0.70 | 1.664 x 10^-6^ | 0.223 | 0.68 | 0.0007418 | 0.69 | 5.959 x 10^-9^ |
| 9 | 133169308 | rs8176681 | T/C | 0.356 | 0.70 | 3.128 x 10^-8^ | 0.381 | 0.60 | 7.518 x 10^-8^ | 0.67 | 8.39 x 10^-14^ |
| 9 | 133171757 | rs514659 | A/C | 0.433 | 1.84 | 4.931 x 10^-22^ | 0.394 | 2.06 | 1.585 x 10^-15^ | 1.91 | 2.225 x 10^-34^ |
| 9 | 133171771 | rs644234 | T/G | 0.454 | 1.70 | 1.724 x 10^-17^ | 0.420 | 1.96 | 1.217 x 10^-13^ | 1.78 | 4.394 x 10^-28^ |
| 9 | 133171909 | rs643434 | G/A | 0.454 | 1.70 | 1.685 x 10^-17^ | 0.420 | 1.96 | 1.29 x 10^-13^ | 1.78 | 4.701 x 10^-28^ |
| 9 | 133172926 | rs545971 | C/T | 0.432 | 1.84 | 4.538 x 10^-22^ | 0.394 | 2.06 | 1.811 x 10^-15^ | 1.91 | 2.589 x 10^-34^ |
| 9 | 133172996 | rs612169 | A/G | 0.432 | 1.85 | 4.354 x 10^-22^ | 0.394 | 2.06 | 1.99 x 10^-15^ | 1.91 | 2.391 x 10^-34^ |
| 9 | 133176218 | rs674302 | T/A | 0.432 | 1.85 | 4.239 x 10^-22^ | 0.394 | 2.05 | 2.05 x 10^-15^ | 1.91 | 2.58 x 10^-34^ |
| 9 | 133178783 | rs505922 | T/C | 0.430 | 1.85 | 1.838 x 10^-22^ | 0.393 | 2.05 | 2.146 x 10^-15^ | 1.92 | 1.386 x 10^-34^ |
| 9 | 133179054 | rs529565 | T/C | 0.430 | 1.85 | 1.845 x 10^-22^ | 0.393 | 2.05 | 2.152 x 10^-15^ | 1.92 | 1.386 x 10^-34^ |
| 9 | 133179276 | rs630014 | G/A | 0.424 | 0.63 | 2.2 x 10^-13^ | 0.445 | 0.60 | 5.637 x 10^-8^ | 0.62 | 2.537 x 10^-19^ |
| 9 | 133180999 | rs568203 | C/A | 0.21 | 0.70 | 1.439 x 10^-6^ | 0.223 | 0.68 | 0.0006175 | 0.69 | 4.825 x 10^-9^ |
| 9 | 133183035 | rs552148 | C/T | 0.21 | 0.70 | 1.416 x 10^-6^ | 0.223 | 0.68 | 0.0006227 | 0.69 | 4.506 x 10^-9^ |
| 9 | 133183429 | rs651007 | C/T | 0.307 | 1.66 | 5.033 x 10^-14^ | 0.268 | 1.77 | 1.425 x 10^-8^ | 1.69 | 8.96 x 10^-21^ |
| 9 | 133183722 | rs579459 | T/C | 0.316 | 1.64 | 2.826 x 10^-14^ | 0.276 | 1.78 | 3.845 x 10^-9^ | 1.68 | 3.336 x 10^-21^ |
| 9 | 133183858 | rs649129 | C/T | 0.316 | 1.64 | 2.915 x 10^-14^ | 0.276 | 1.78 | 3.755 x 10^-9^ | 1.68 | 3.557 x 10^-21^ |
| 9 | 133184421 | rs495828 | G/T | 0.316 | 1.64 | 3.015 x 10^-14^ | 0.276 | 1.78 | 3.734 x 10^-9^ | 1.68 | 4.002 x 10^-21^ |
| 9 | 133184554 | rs9411489 | C/T | 0.295 | 1.82 | 5.025 x 10^-19^ | 0.250 | 1.95 | 2.937 x 10^-11^ | 1.86 | 6.029 x 10^-28^ |
| 9 | 133184897 | rs2157772 | G/A | 0.209 | 0.68 | 5.83 x 10^-7^ | 0.224 | 0.68 | 0.0006596 | 0.68 | 2.159 x 10^-9^ |
| 9 | 133184998 | rs633862 | T/C | 0.378 | 0.62 | 6.801 x 10^-14^ | 0.411 | 0.58 | 5.981 x 10^-9^ | 0.61 | 8.102 x 10^-21^ |
| 9 | 133185143 | rs502361 | G/C | 0.209 | 0.68 | 5.718 x 10^-7^ | 0.224 | 0.68 | 0.0006743 | 0.68 | 2.224 x 10^-9^ |
| 9 | 133186687 | rs7865877 | G/A | 0.490 | 1.42 | 5.05 x 10^-9^ | 0.447 | 1.38 | 0.0002307 | 1.41 | 5.537 x 10^-12^ |
| 9 | 133268953 | rs3758348 | G/C | 0.202 | 1.90 | 1.728 x 10^-11^ | 0.183 | 1.99 | 2.38 x 10^-7^ | 1.93 | 3.855 x 10^-17^ |
| 9 | 133353308 | rs4962153 | G/A | 0.177 | 2.49 | 7.001 x 10^-11^ | 0.178 | 2.22 | 7.719 x 10^-6^ | 2.38 | 5.455 x 10^-15^ |
| 9 | 133355802 | rs739468 | G/T | 0.177 | 2.51 | 7.593 x 10^-11^ | 0.177 | 2.25 | 7.842 x 10^-6^ | 2.40 | 5.376 x 10^-15^ |
